# Structural basis for selective recognition of acyl chains by the membrane-associated acyltransferase PatA

**DOI:** 10.1038/ncomms10906

**Published:** 2016-03-11

**Authors:** David Albesa-Jové, Zuzana Svetlíková, Montse Tersa, Enea Sancho-Vaello, Ana Carreras-González, Pascal Bonnet, Pedro Arrasate, Ander Eguskiza, Shiva K. Angala, Javier O. Cifuente, Jana Korduláková, Mary Jackson, Katarína Mikušová, Marcelo E. Guerin

**Affiliations:** 1 Unidad de Biofísica, Consejo Superior de Investigaciones Científicas—Universidad del País Vasco/Euskal Herriko Unibertsitatea (CSIC,UPV/EHU), Barrio Sarriena s/n, Leioa, 48940 Bizkaia, Spain; 2 Departamento de Bioquímica, Universidad del País Vasco, Leioa, 48940 Bizkaia, Spain; 3 IKERBASQUE, Basque Foundation for Science, 48013 Bilbao, Spain; 4 Department of Biochemistry, Faculty of Natural Sciences, Comenius University in Bratislava, 84215 Bratislava, Slovakia; 5 Université d'Orléans et CNRS, ICOA, UMR 7311, F-45067 Orléans, France; 6 Mycobacteria Research Laboratories, Department of Microbiology, Immunology and Pathology, Colorado State University, Fort Collins, Colorado 80523-1682, USA; 7 Structural Biology Unit, CIC bioGUNE, Bizkaia Technology Park, 48160 Derio, Spain

## Abstract

The biosynthesis of phospholipids and glycolipids are critical pathways for virtually all cell membranes. PatA is an essential membrane associated acyltransferase involved in the biosynthesis of mycobacterial phosphatidyl-*myo*-inositol mannosides (PIMs). The enzyme transfers a palmitoyl moiety from palmitoyl–CoA to the 6-position of the mannose ring linked to 2-position of inositol in PIM_1_/PIM_2_. We report here the crystal structures of PatA from *Mycobacterium smegmatis* in the presence of its naturally occurring acyl donor palmitate and a nonhydrolyzable palmitoyl–CoA analog. The structures reveal an α/β architecture, with the acyl chain deeply buried into a hydrophobic pocket that runs perpendicular to a long groove where the active site is located. Enzyme catalysis is mediated by an unprecedented charge relay system, which markedly diverges from the canonical HX_4_D motif. Our studies establish the mechanistic basis of substrate/membrane recognition and catalysis for an important family of acyltransferases, providing exciting possibilities for inhibitor design.

## Abstract (web summary)

PatA is a membrane-associated acyltransferase that is essential for the biosynthesis of mycobacterial glycolipids. Here, Albesa-Jové *et al*. describe structures of PatA from *Mycobacterium smegmatis* in complex with acyl donors and show that catalysis occurs by an unusual charge-relay mechanism.

## Graphical abstract

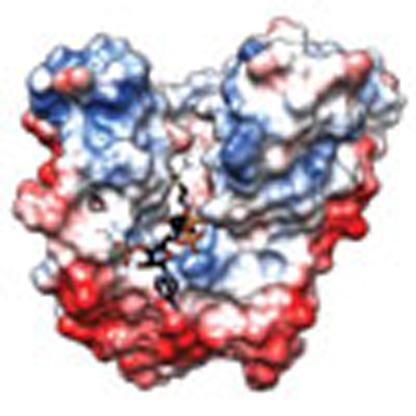

Long-chain fatty acids play a central role in a variety of important biological processes in all living organisms. They are prominent constituents of biological membranes, mainly in the form of phospholipids, allowing cells to be functionally constituted and differentiated from the environment^1^. Long-chain fatty acids are used as energy storage and metabolic intermediates as well as being modulators of signal transduction pathways^2^,^3^. Moreover, the attachment of fatty acids to proteins and glycans generates a significant amount of structural diversity in biological systems^4^. This structural information is particularly apparent in molecular recognition events including cell–cell interactions during critical steps of development and host–pathogen interactions. Fatty acids are usually activated for subsequent reactions by esterification of their carboxyl groups with the thiol group of coenzyme A (CoA) or that of the acyl carrier protein (ACP), yielding acyl-thioesters^5^. Acyltransferases are key enzymes that catalyse the transfer of activated acyl chains to acceptor molecules of different chemical structure and complexity^6^. Importantly, acyltransferases are involved in the biosynthesis of triacylglycerols and a diverse group of naturally occurring polyesters composed of 3-hydroxyalkanoic acids, the most important storage lipids found in eukaryotes and prokaryotes, respectively^5^. Moreover, acyltransferases participate in the biosynthesis of the lipid A moiety of lipopolysaccharide, modulating virulence of some Gram-negative human pathogens^7^,^8^. Therefore, the understanding of the mechanism of action for these enzymes at the molecular level, particularly those firmly associated to the lipid bilayer, represents a major challenge.

The phosphatidyl-*myo*-inositol mannosides (PIMs) are glycolipids of exceptional chemical structure found in abundant quantities in the inner and outer membranes of the cell envelope of all *Mycobacterium* species^9^. PIMs are based on a phosphatidyl-*myo*-inositol (PI) lipid anchor carrying one to six Man*p* residues and up to four acyl chains, with tri- and tetra-acylated phosphatidyl-*myo*-inositol dimannoside (PIM_2_) and phosphatidyl-*myo*-inositol hexamannoside (PIM_6_) as the predominant species (Fig. 1a)^10^,^11^. PIMs are thought to be the precursors of the two major mycobacterial lipoglycans, lipomannan and lipoarabinomannan (LAM). PIMs, lipomannan and LAM are considered not only essential structural components of the mycobacterial cell envelope^10^,^11^,^12^,^13^ but also important molecules implicated in host–pathogen interactions in the course of tuberculosis and leprosy^14^,^15^. The biosynthesis of PIMs is initiated by the phosphatidyl-*myo*-inositol mannosyltransferase PimA (Rv2610c in *Mycobacterium tuberculosis* (*M. tuberculosis*) H37Rv), which catalyses the transfer of Man*p* residue from GDP-Man to the 2-position of the *myo*-inositol ring of PI, to form phosphatidyl-*myo*-inositol monomannoside (PIM_1_) on the cytoplasmic face of the plasma membrane (Fig. 1b)^12^,^16^. PimA was found to be essential for *Mycobacterium smegmatis* (*M. smegmatis*) mc^2^155 and *M. tuberculosis* growth *in vitro* and *in vivo*^12^,^17^. The second mannosylation step involves the action of another essential enzyme PimB (Rv2188c in *M. tuberculosis* H37Rv), which transfers a Man*p* residue to the 6-position of the *myo*-inositol ring of PIM_1_ to form PIM_2_ (refs 17, 18). Both PIM_1_ and PIM_2_ can be acylated with palmitate at position 6 of the Man*p* residue transferred by PimA by the acyltransferase PatA (*P*hosphatidyl-*myo*-inositol mannosides *A*cyl*T*ransferase *A*; Rv2611c in *M. tuberculosis* H37Rv), to form Ac_1_PIM_1_ and Ac_1_PIM_2_, respectively^19^,^20^,^21^. This enzyme has been proved (i) to be important for the optimal growth of *M. smegmatis* mc^2^155 and (ii) an essential enzyme for the growth of *M. tuberculosis in vitro*^10^,^21^. Two models were originally proposed for the biosynthesis of Ac_1_PIM_2_ in mycobacteria. In the first model, PI is mannosylated to form PIM_1_. PIM_1_ is then mannosylated to form PIM_2_, which is acylated to form Ac_1_PIM_2_. In the second model, PIM_1_ is first acylated to Ac_1_PIM_1_ and then mannosylated to Ac_1_PIM_2_. Recent evidence indicates that although both pathways might co-exist in mycobacteria, the sequence of events PI→PIM_1_→PIM_2_→Ac_1_PIM_2_ is favoured (Fig. 1b)^13^. Finally, Ac_1_PIM_2_ can be further acylated on position 3 of the *myo*-inositol ring to form Ac_2_PIM_2_. However, this acyltransferase as well as most of the mannosyltransferases that catalyse the formation of higher PIMs still remains to be identified^10^.

During recent years major efforts have been made to understand the early steps of the PIM biosynthetic pathway at the molecular level. In that sense, the crystal structures of the mannosyltransferases PimA and PimB were reported, showing the typical organization and catalytic machinery of GT-B glycosyltransferases^22^,^23^,^24^,^25^. However, to date, no structural information is available for PatA, an enzyme that is a member of a large family of acyltransferases for which the molecular mechanism of substrate recognition and catalysis remains not well understood^5^,^7^,^26^,^27^. Here, X-ray crystallography, site-directed mutagenesis and enzymatic activity data are used to define the three-dimensional structure, acceptor/donor substrate binding, membrane binding and catalytic mechanism of PatA from *M. smegmatis*. Our results reveal that the enzyme has an α/β architecture and shares with other important eukaryotic/prokaryotic acyltransferases an acid/base catalytic mechanism involving conserved histidine and aspartic/glutamic acid residues.

## Results

### Overall structure of PatA

**Figure 2:**
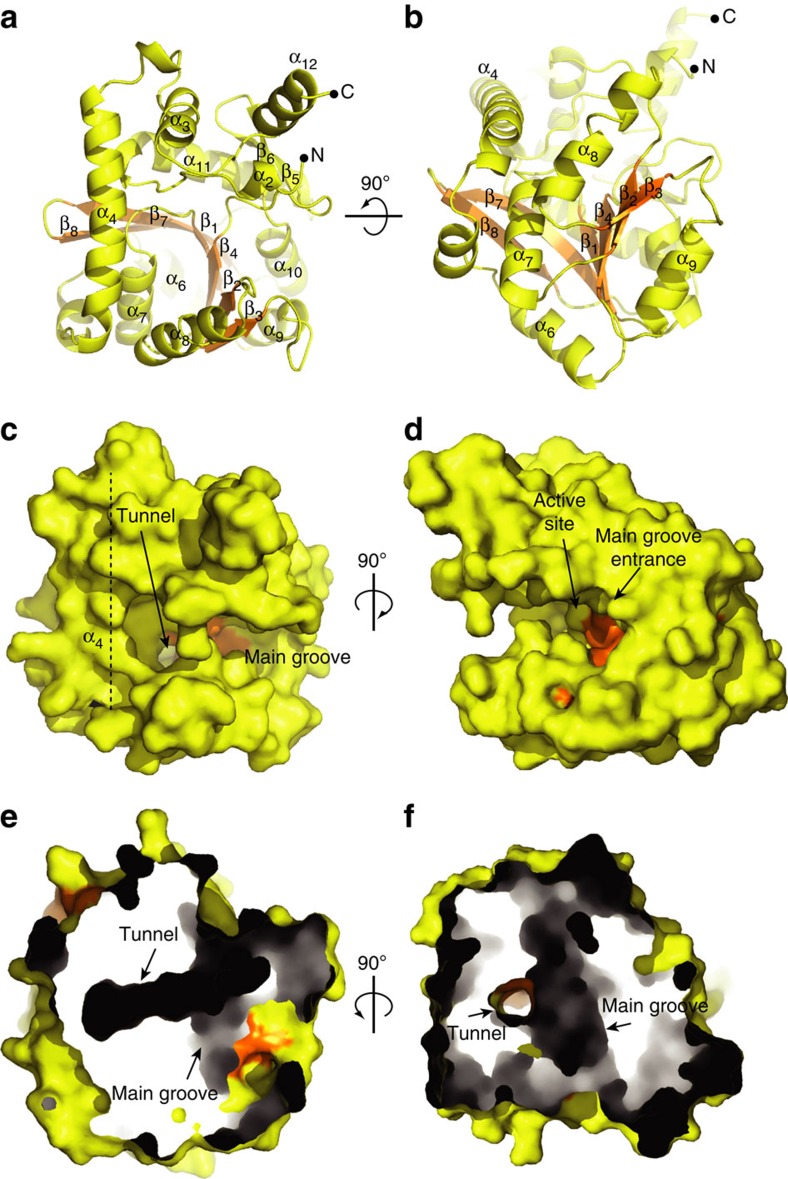
Overall structure of PatA. (**a**,**b**) Cartoon representation showing the general fold and secondary structure organization of PatA. Secondary structure elements are labelled. The central core β-sheet is shown in orange. (**c**,**d**) Surface representation of PatA showing the location of the main groove and the active site. The groove entrance is flanked by two important α-helices, α_11_–α_12_. The groove ends up into a cavity mainly flanked by α_4_. (**e**,**f**) The main groove runs perpendicular to a hydrophobic tunnel, which is deeply buried into the core of PatA.

The crystal structure of PatA from *M. smegmatis* was solved using single-wavelength anomalous dispersion with a K_2_PtCl_4_ derivative at 2.06 Å resolution in *C* 2 space group (PatA–C16-1). Two other crystal forms were obtained in *P* 2_1_ (PatA–C16-2) and *P* 4_2_2_1_2 (PatA–C16-3) space groups, and the corresponding crystal structures solved at 2.90 and 2.43 Å resolution, respectively, by using molecular replacement methods (see Methods and Supplementary Information for details; Supplementary Fig. 1; Supplementary Tables 1 and 2). The high quality of the electron density maps allowed the trace of residues 41 to 302 (PatA–C16-1), 48 to 295 (PatA–C16-2) and 48 to 303 (PatA–C16-3; Supplementary Fig. 2). We have decided to use the PatA–C16-1 crystal form for our description since it displays the highest resolution. A close inspection of the three crystal structures revealed that the protein crystallized as a monomer, displaying a high degree of structural flexibility in the N- and C-terminal regions (coloured in green in Supplementary Fig. 3; root-mean-square deviation (r.m.s.d.) value of 1.6 and 8.4 Å for Cα atoms in 41–47 and 288–302 residue ranges for the N and C terminus, respectively). The central core of PatA consists of a six-stranded β-sheet with topology β_1_−β_2_−β_3_−β_4_−β_7_−β_8_ (β_8_ is antiparallel) surrounded by α-helices, with an overall size of 45 × 40 × 40 Å (Fig. 2a,b). A long and open groove that runs parallel to the protein surface contains the active site. This groove is flanked by α_4_, β_2_, α_8_, β_4_, α_9_, α_10_ and the connecting loops β_1_–α_7_ (residues 124–130), β_2_–α_8_ (residues 148–154), β_3_–α_9_ (residues 173–181), β_4_–β_5_ (residues 198–208), β_6_–α_10_ (residues 219–221) and α_11_–α_12_ (residues 282–291; Fig. 2c,d). Strikingly, the groove also displays a narrow and deep, mostly hydrophobic tunnel that runs perpendicular from its floor to the central core of the protein (Fig. 2e,f). The walls of the tunnel comprise the entire central β-sheet, two α-helices α_7_ and α_8_, and the connecting loops β_1_–α_7_ (residues 124–130) and α_8_–β_6_ (residues 167–170). The cavity extends entirely through the core and is closed at the bottom by β_8_, the connecting loop α_5_–α_6_ and part of the α_6_ (Fig. 2b,e). Interestingly, the entrance of the hydrophobic pocket displays several charged residues compatible to interact with a buried carboxylate group of a fatty acid moiety, strongly suggesting the pocket might play a role in the donor substrate binding.

### Membrane association

PatA catalyses an essential step in the biosynthesis of PIMs in *M. tuberculosis*^10^. The enzyme transfers a palmitoyl moiety from palmitoyl–CoA to the 6-position of the Man*p* ring linked to 2-position of *myo*-inositol in PIM_1_ or PIM_2_. A close interaction of the enzyme with the cytosolic face of the mycobacterial plasma membrane might be a strict requirement for PIM_1_ or PIM_2_ modification by PatA. Supporting this notion, PatA was found to co-localize with the mycobacterial membrane fraction^19^,^28^. Analysis of the amino-acid sequence of the enzyme revealed the lack of a signal peptide or hydrophobic transmembrane segments, suggesting that PatA associates to only one side of the lipid bilayer, a typical feature of peripheral and monotopic membrane proteins^24^,^29^. To perform their biochemical functions, these proteins very often display a high content of solvent exposed positively charged residues in the form of amphiphatic helices, promoting membrane surface interaction with anionic phospholipids^30^. Protein–membrane interaction appears to be mediated following different thermodynamic steps: (i) peptide binding is initiated by the electrostatic attraction of the positively charged residues to the anionic membrane, (ii) most likely followed by the transition of the peptide into the plane of binding and (iii) a change of the conformation of the bound peptide^31^. The penetration of the protein depends on the chemical nature of the lipids and peptides involved and also on the mechanistic nature of the processes involved, in which both location and timing of membrane association can be tightly controlled. The electrostatic surface potential of PatA revealed a clear solvent-exposed area adjacent to the major groove that contain several hydrophobic patches interspersed with clusters of positively charged residues^32^. Specifically, this region comprises the α_2_, the amphiphatic helices α_3_, α_4_ and α_8_, the connecting loop β_2_–α_8_ (residues 148–154), and likely the α_1_ and α_12_ helices located at the N and C terminus of the protein (Supplementary Fig. 4). Interestingly, the opposite side of PatA displays a negatively charged surface, which would generate a significant electrostatic repulsion with the lipid bilayer. Thus, the polar character of PatA certainly contributes to determine the correct orientation of the enzyme into the membrane (Supplementary Fig. 4).

### The acyl–CoA-binding site

**Figure 3:**
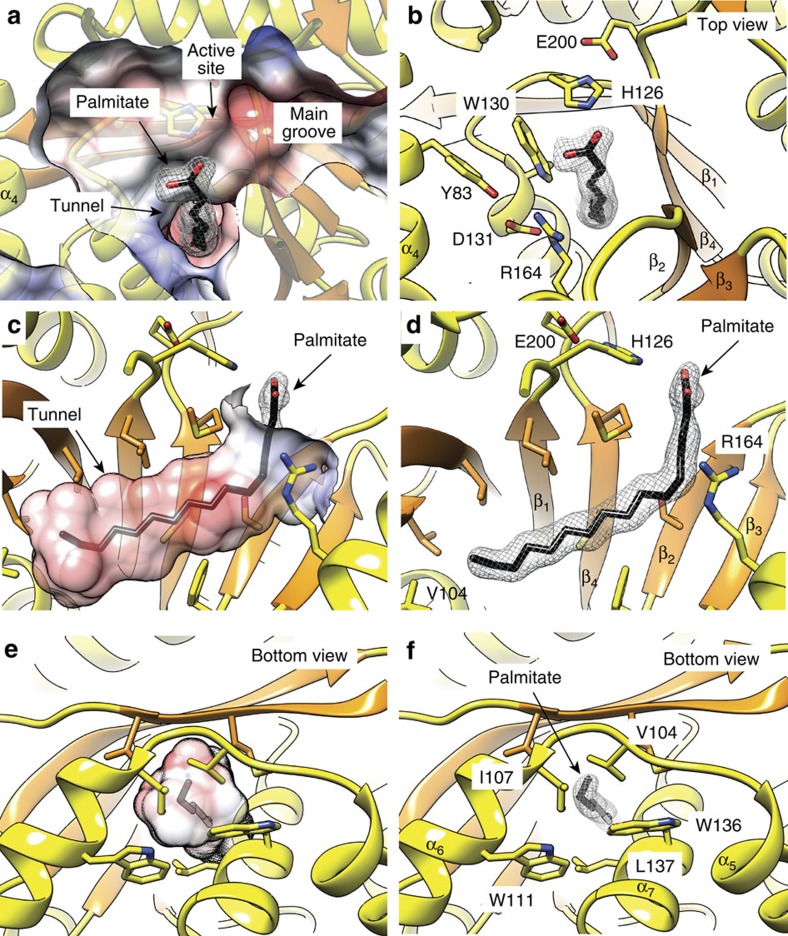
The palmitate binding site of PatA. (**a**–**d**) Four views of PatA–C16-1 crystal structure showing the palmitate chain deeply buried into the hydrophobic tunnel. (**e**,**f**) Two views of PatA showing the cap which closes the bottom of the hydrophobic tunnel. The 2mFo-DFc electron density map countered at 1 σ for the palmitate ligand is shown.

Strikingly, one molecule of palmitic acid was unambiguously identified in the difference electron density maps of PatA–C16-1, PatA–C16-2 and PatA–C16-3 crystal structures (Fig. 3a–d). We believe that acyl molecule is associated to the enzyme due to the hydrolysis of palmitoyl–CoA during the isolation and purification of PatA from *M. smegmatis* mc^2^155. Supporting this notion, the chemical structure of Ac_1_PIM_2_ was clearly established by using a combination of analytical techniques including mass spectrometry and two-dimensional NMR^20^. The major acyl form observed, corresponded to PIM_2_ with the glycerol moiety being di-acylated by C_16_/C_19_ and the mannose residue transferred by PimA bearing a C_16_. This structural profile was also identified in its hexamannosylated derivative Ac_1_PIM_6_ (ref. 21). The acyl chain is deeply buried into the hydrophobic pocket, and oriented with the carboxylate group facing the groove and the acyl tail extending into the globular core of the monomer (Fig. 3). The residues that contact the bound palmitic acid are highly conserved in the PatA mycobacterial homologues (Supplementary Fig. 5). Interestingly, the comparison of the three crystal structures revealed conformational flexibility in the carboxylate moiety of the palmitate (Supplementary Fig. 3), located in close proximity with the lateral chain of H126. The R164 guanidinium group engages the side chain of Y83 and D131. This tryptophan residue, together with M198, makes an important van der Waals interaction with the acyl chain. The acyl chain undergoes a kink at position C6 and terminates in a pocket mainly formed by hydrophobic residues, including L122 and L124 (β_1_), A133, W136 and L137 (α_7_), F144 (α_7_–β_2_ loop), T146 (β_2_), F169 (α_8_–β_3_ loop), F235 and V237 (β_7_), M248 and V250 (β_8_), and two cysteine residues C196 (β_4_) and C239 (β_7_). Interestingly, the connecting loop α_5_–α_6_ (residues 101–106) and part of α_6_ form a flexible and mostly hydrophobic cap that closes the bottom of the cavity, suggesting that PatA might be able to discriminate the length of the acyl chain groups (Fig. 3e,f). It is worth noting that Ac_1_PIM_2_ was the main product formed in the reaction when endogenous or crude mycobacterial phospholipids from *Mycobacterium phlei* were used as the lipid acceptors and a series of acyl–CoA derivatives of fatty acids were used as ^14^C-labelled donor substrates^33^,^34^. Palmitoyl–CoA (C16:0) gave higher incorporation than myristyl–CoA (C14:0). Interestingly, the oleyl–CoA (C18:1) was a much better substrate than the saturated counterpart stearyl–CoA (C18:0). Finally, the tuberculostearic acid (C19:1) had a low specific activity and the small incorporation of label that was observed may be not significant^34^.

**Figure 4:**
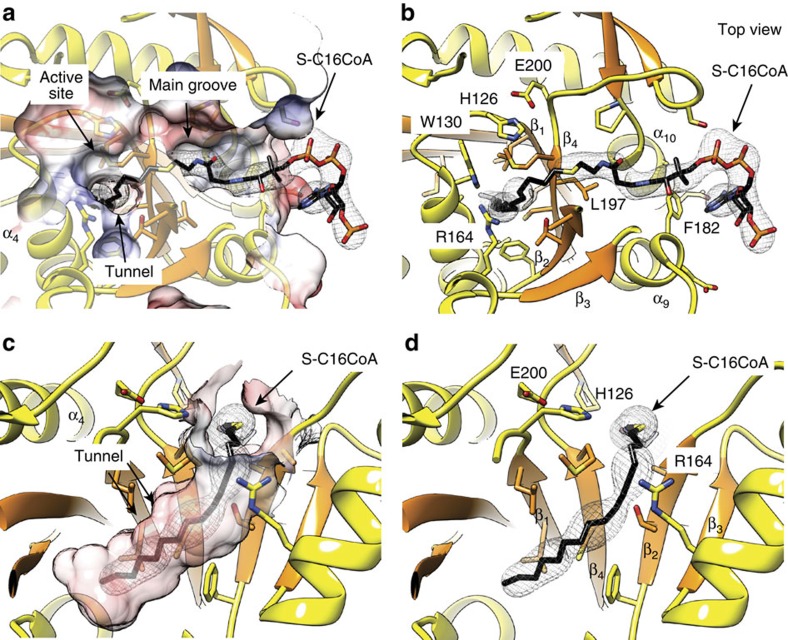
The palmitoyl–CoA binding site of PatA. (**a**–**d**) Four views of PatA-S-C16CoA crystal structure showing the binding mechanism of S-C16CoA. The palmitoyl moiety is deeply buried into the hydrophobic tunnel, whereas the coenzyme A moiety extends outwards through the side of the main groove. The 2mFo-DFc electron density map countered at 1 σ for the palmitate ligand is shown.

**Figure 5:**
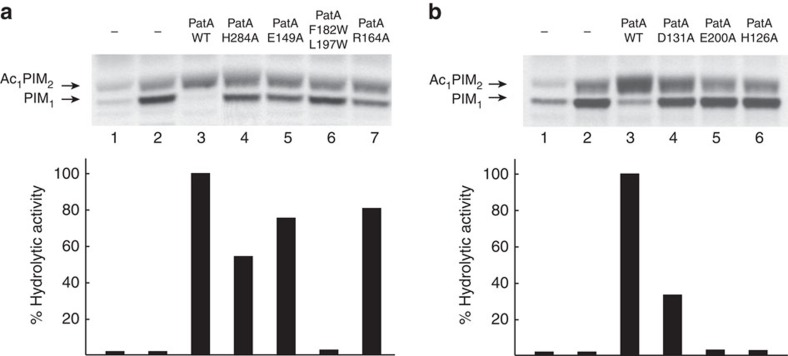
Enzymatic activity of selected PatA variants. (**a**) In the upper panel, the acyltransferase activity of purified PatA and variants involved in substrate binding are shown (see Supplementary Fig. 9 for duplicates). Reaction mixtures contained crude membranes from *M. smegmatis* mc^2^155 and GDP-[^14^C]-mannose as a tracer (lane 1), supplemented with PimA (lanes 2–7) and palmitoyl–CoA (lanes 3–7) and purified PatA (lane 3), PatA-H284A (lane 4), PatA-E149A (lane 5), double mutant PatA-F182W/L197W (lane 6) and PatA-R164A (lane 7). The lipids were extracted from reaction mixtures and analysed by TLC and autoradiography as described in Methods section. In the lower panel, the hydrolytic activity against palmitoyl–CoA is shown. (**b**) In the upper panel, the acyltransferase activity of purified PatA and variants involved in catalysis are shown (see Supplementary Fig. 9 for duplicates). Reaction mixtures contained crude membranes from *M. smegmatis* mc^2^155 and GDP-[^14^C]-mannose as a tracer (lane 1), supplemented with PimA (lane 2–6) and palmitoyl–CoA (lanes 3–6) and purified PatA (lane 3), PatA-D131A (lane 4), PatA-E200A (lane 5) and PatA-H126A (lane 6). In the lower panel, the hydrolytic activity against palmitoyl–CoA is shown as determined by spectrophotometric analysis. All enzymatic activities measurements were determined in duplicates (Supplementary Fig. 9)

How does PatA recognize CoA? To this end, the crystal structure of PatA in complex with *S*-hexadecyl Coenzyme A (S-C16CoA), a nonhydrolyzable analogue of palmitoyl–CoA, was solved at 3.28 Å resolution in *P* 2_1_ space group (PatA–S-C16CoA; Supplementary Table 1; Fig. 4). The acyl chain of S-C16CoA is localized into the hydrophobic tunnel, and superimposes very well with the acyl chain moiety of palmitate observed in the PatA–C16-1, PatA–C16-2 and PatA–C16-3 complexes. It is worth noting that the position of the carboxylate group of palmitate is different to that observed for the thioether in the PatA–S-C16CoA complex. The 4-phosphopantetheinate moiety of S-C16CoA is clearly defined in the electron density map, and located at the entrance of the main groove, in close contact with a highly conserved region flanked by the β_2_–α_8_ (residues 149–153), β_3_–α_9_ (residues 174–180) and β_4_–β_5_ (residues 199–207) loops, and two alpha helices, α_9_ (residues 181–190) and α_10_ (residues 221–230; Supplementary Fig. 6). The adenosine 3′,5′-diphosphate (3′,5′-ADP) moiety of the ligand (disordered in other monomers of the asymmetric unit) sticks out from the globular core and is exposed to the bulk solvent, as observed in other acyl–CoA modifying enzymes (Supplementary Fig. 7)^35^. The approximate volume of the palmitoyl–CoA-binding pocket was *ca.* 2,801 Å^3^(ref. 36) To further validate the model, we designed site-directed mutations predicted to impair the palmitoyl–CoA interaction with PatA. Thus, the double substitution F182W/L197W would block the groove region of PatA, hindering the formation of the complex (Fig. 4b and Supplementary Fig. 8). As depicted in Fig. 5a, the PatA F182W/L197W variant could not (i) transfer a palmitoyl moiety to PIM_2_, or (ii) hydrolyze palmitoyl–CoA, supporting the proposed model (Supplementary Fig. 9).

### The phosphatidylinositol mannosides binding site

**Figure 6:**
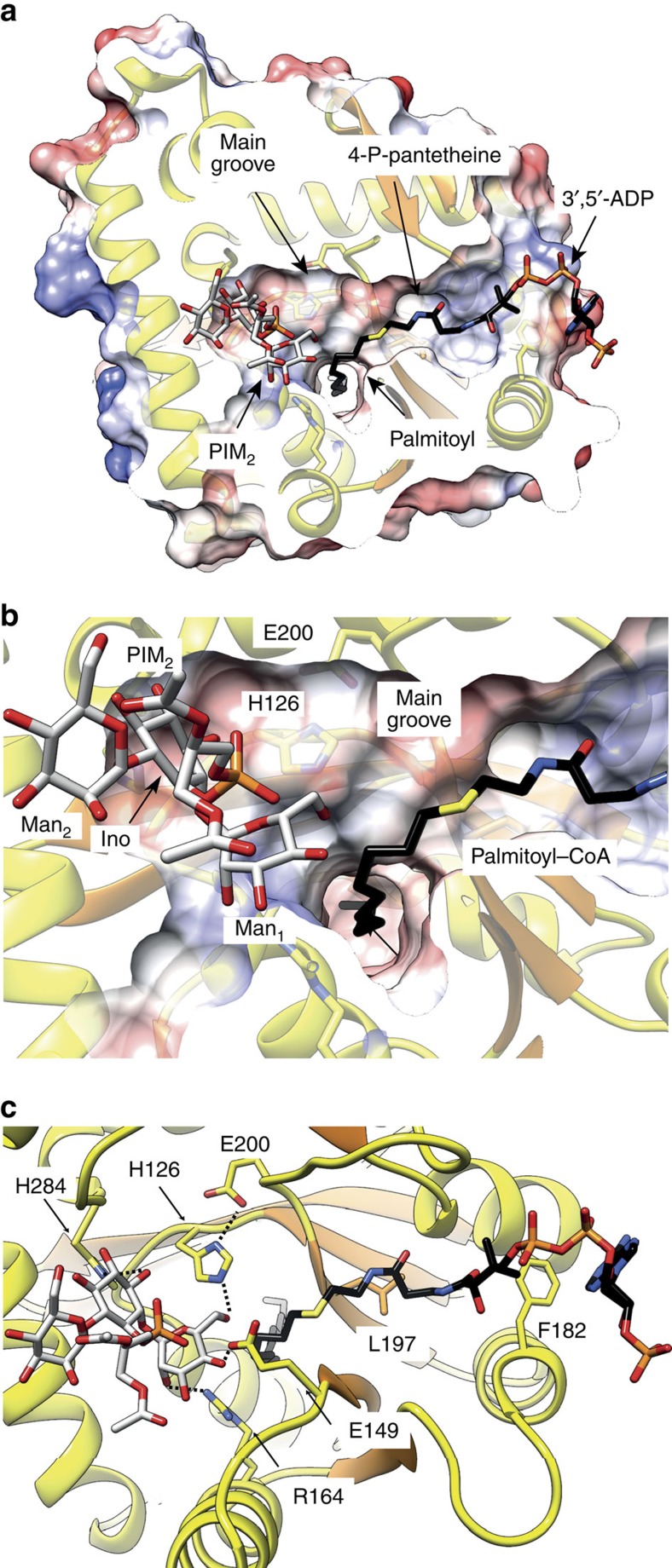
The phosphatidylinositol mannosides binding site. (**a**,**b**) Two views showing the docking calculations in which PIM_2_ attaches to the end side of the main groove, close to the hydrophobic tunnel. (**c**) Close view of the binding site, showing the location of predicted residues involved in PIM_2_ interaction that have been mutated (H126, E149, R164, H284) along with mutated residues involved in CoA binding (L197 and F182).

Although we were unable to co-crystallize PatA in complex with Ac_1_PIM_1_/Ac_2_PIM_1_, Ac_1_PIM_2_/Ac_2_PIM_2_ or their deacylated analogs, the three-dimensional structure suggests the possible binding mode for the polar head of PIM_1_ or PIM_2_ acceptor substrates within the active site. Docking calculations placed the polar head of PIM_2_, the better substrate of PatA making important interactions within a region located at the end of the main groove and comprising helices α_4_ and α_8_, and the connecting loops β_1_–α_7_ (residues 83–90), β_2_–α_8_ (residues 148–154) and α_11_–α_12_ (residues 282–291; Fig. 6)^13^,^28^. As a consequence, the O6 atom of the Man*p* ring linked to position 2 of *myo*-inositol in PIM_2_ is predicted to be positioned favourably for activation by H126 and to receive the palmitate group from palmitoyl–CoA (Fig. 6; distance H126 NE2 atom to Man*p* O6 atom is 2.8 Å). The model also predicts an important role of residues E149, R164 and H284 to bind PIM_2_ in the active site. In the model, E149 OE1 atom is found at 2.9 Å of the Man*p* residue O4 atom and R164 NH2 atom is placed at 3.1 Å of the O2 atom of the Man*p* ring, whereas H284 ND1 atom is at only 2.5 Å of *myo*-inositol O3 atom. The *myo*-inositol moiety and the Man*p* ring linked to position 6 of *myo*-inositol in PIM_2_ also interact with Y80 and Q287. The approximate volume of the PIM_2_-binding pocket was *ca.* 1741 Å^3^ (ref. 36). Interestingly, docking calculations put the *myo*-inositol and Man*p* moieties of PIM_1_ in an equivalent position to that observed for PIM_2_, leaving free space in the pocket, a fact that might account for the acceptor substrate specificity of PatA (Supplementary Fig. 10). To experimentally validate the proposed model, we designed three single-point substitution, E149A, R164A and H284A, predicted to impact the PIM_2_ interaction with PatA (Fig. 6c; Supplementary Fig. 8). As depicted in Fig. 5a, the transferase activity of all PatA variants was severely compromised, nevertheless preserving the capability to hydrolyse palmitoyl–CoA (Supplementary Fig. 9).

### Structural similarity with other acyltransferases

**Figure 7:**
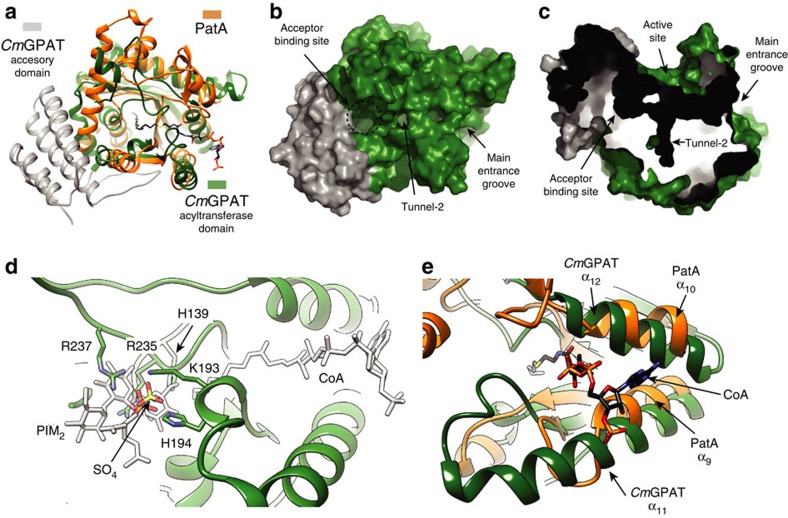
Structural similarities between PatA and *Cm*GPAT. (**a**) Structural superposition of PatA and *Cm*GPAT. Secondary structure elements are labelled. The localization of the fatty acid and acceptor binding sites in *Cm*GPAT are indicated. (**b**,**c**) The active site of *Cm*GPAT is located into a main groove, with a hydrophobic tunnel running perpendicular and deeply buried into the core of the enzyme. (**d**) Structural superposition of the proposed acceptor binding site in PatA and *Cm*GPAT, showing donor and acceptor PatA substrates for spatial reference in white colour. (**e**) Structural superposition of the CoA binding site in PatA and *Cm*GPAT.

Structure homologue search using DALI server revealed only one protein with significant structural similarity, that of the glycerol-3-phosphate acyltransferase from *Cucurbita moschata* (*Cm*GPAT; pdb codes 1IUQ and 1K30; *Z*-score of 8.9; r.m.s.d. value of 3.9 Å for 164 aligned residues; Fig. 7a)^37^,^38^. *Cm*GPAT catalyses the transfer of an acyl chain either from acyl–acyl-carrier protein (acyl–ACP) or acyl–CoA, to the *sn*-1 position of glycerol-3-phosphate, to form 1-acylglycerol-3-phosphate^39^. Importantly, *Cm*GPAT is able to use palmitoyl–CoA as a donor substrate, as PatA does, using a bi–bi-ordered mechanism^40^. *Cm*GPAT belongs to a large family of glycerol-3-phosphate acyltransferases (GPAT), which are critical in the biosynthesis and regulation of phospholipids composition in prokaryotic and eukaryotic cells^41^,^42^,^43^. The structure of *Cm*GPAT is composed of two domains: (i) a helical domain comprising the first 78 residues of the protein displays a four-helix bundle architecture of unknown function, and (ii) an α/β domain, consisting of a nine-stranded continuous β-sheet surrounded by 11 α-helices (Fig. 7a). As depicted in Fig. 7a, the central β-sheet of *Cm*GPAT superimposes well with that observed in PatA, with the exception of its outermost strands β_1_, β_2_ and β_9_, which are missing in PatA (see also Supplementary Fig. 11).

A combination of site-directed mutagenesis and activity measurements provided experimental support on the location of the acceptor-binding site in *Cm*GPAT (Fig. 7b–d)^44^. The replacement of R235, R237 and K193 by serine resulted in inactive enzymes. However, the *Cm*GPAT variants retained the ability to bind stoichiometric quantities of acyl–ACPs, consistent with the location of these residues in the positively charged acceptor-binding pocket, and in close proximity to the catalytic HX_4_D motif^37^,^38^,^44^. Interestingly, the suggested PIM_1_/PIM_2_-binding site in PatA is located in the same region of the glycerol-3-phosphate-binding site in *Cm*GPAT (Fig. 7d). A sulfate ion observed in the crystal structure of *Cm*GPAT superimposed very well with the phosphate moiety of the inositol ring of PIM_1_ or PIM_2_ (Fig. 7d). It is worth noting that, as acceptors exhibit a marked diversity of chemical structures compared with acyl–CoA or acyl–ACP donors, the acceptor-binding sites reflect this variability by showing different rearrangements of secondary structural elements (Fig. 7d).

The location of the acyl–CoA and acyl–ACP-binding site in *Cm*GPAT has been a matter of strong debate^37^,^38^. On the basis of sequence conservation analysis, a structural model in which the acyl chain runs over the entrance of the main groove of the protein was first proposed^37^. Molecular surface calculations revealed the existence of three tunnels with sufficient space to accommodate the acyl chain of palmitoyl–CoA^45^. Furthermore, enzymatic analysis of chimeric *Cm*GPAT and *Spinacea oleracea* GPAT revealed that a region comprising residues 128–187 is important for acyl–CoA selectivity. This region completely covered the narrowest and most hydrophobic of the three tunnels, tunnel-2 (Fig. 7b,c), which was proposed to be involved in fatty-acid recognition^38^. Strikingly, the acyl-binding pocket identified in the crystal structures of the palmitoyl–PatA and PatA-S-C16CoA complexes superimposed very well with tunnel-2 of *Cm*GPAT (Supplementary Fig. 11). On the basis of the experimental location of the S-C16CoA in PatA, a palmitoyl–CoA molecule was fitted into tunnel-2 and subjected to energy minimization (Fig. 7d,e; Supplementary Fig. 11; see Methods for details). First, the palmitate moiety accommodates into the hydrophobic tunnel. The walls of the tunnel are covered with mainly conserved, hydrophobic and aromatic residues including L135 (β_3_), P145 and I148 (α_7_), I159 (α_8_), T163 (α_8_–β_4_ loop), F165 (β_4_), F180 (α_10_), L186 (β_5_), L226 and W228 (β_6_), L274 and L276 (β_7_) and L305, with F120 and I123 (α_6_) making up the cap. Interestingly, the CoA-binding site observed in PatA is also conserved in *Cm*GPAT. Specifically, the acyl donor is located in the region corresponding to the entrance of the main groove (Fig. 7c,e). The two α-helices α_9_ and α_10_, flanking the entrance of acyl–CoA to the main groove of PatA, are structurally equivalent to α_11_ (residues 201–222) and α_12_ (residues 251–265) in *Cm*GPAT (Fig. 7e). The substitution of L261 (α_12_) by an aromatic residue, which is located at the interface of α_11_–α_12_ helices, caused major changes in the selectivity of *Cm*GPAT for acyl–ACP derivatives^44^. Overall, major secondary structural elements are structurally preserved across both families of acyltransferases supporting a common binding mode for palmitoyl–CoA.

### The catalytic mechanism of PatA

**Figure 8:**
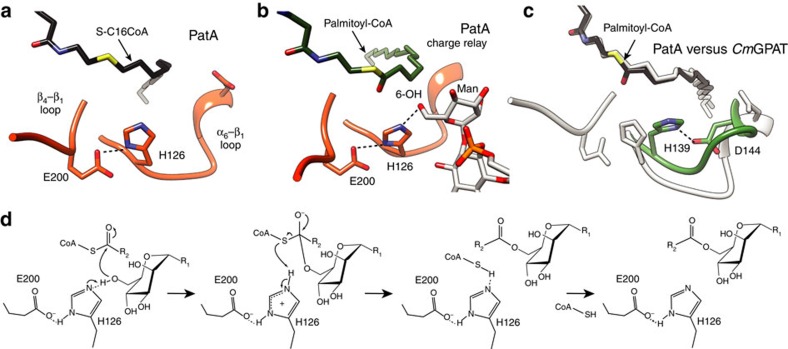
Catalytic mechanism of PatA. (**a**) Active site configuration of PatA, as visualized in the crystal structure in complex with S-C16CoA. (PatA–S-C16CoA) (**b**) Active site configuration of PatA as visualized in the docking of palmitoyl–CoA and PIM_2_ (the atomic coordinates of palmitoyl–CoA were derived from S-C16CoA following *in silico* addition of an oxygen atom at postion C16 of S-C16CoA). The charged relay system formed between E200, H126 and the 6-OH of the mannose linked to the 2-position of inositol (ino) is highlighted. (**c**) Structural superposition of the catalytic site of PatA (grey) and *Cm*GPAT (green) with palmityol–CoA docked into *Cm*GPAT. (**d**) Proposed catalytic mechanism for PatA.

Analysis of the cleft running over the surface of PatA revealed a catalytic site reminiscent to that observed in the serine protease family of enzymes. In serine proteases, the cleavage of the peptide bond is mediated by nucleophilic attack of the serine hydroxyl group on the scissile carbonyl bond. The active site comprises a catalytic triad consisting of the Oγ atom of the serine, the imidazole ring of a histidine, and the carboxylate group of an aspartic/glutamic acid, involved in a charge relay system that increases the nucleophilicity of the serine hydroxyl and modulates the pKa of the central histidine as a general base or acid during the catalytic cycle^45^. In all crystal structures of palmitoyl–PatA and PatA–S-C16CoA complexes, the carboxylate OE2 oxygen atom of E200 was found at 2.8 Å of the ND1 nitrogen atom of the aromatic imidazole ring of the invariant H126 (Fig. 8a). It is worth noting that when the individual palmitate molecules observed in the three crystal structures of palmitoyl–PatA were superimposed, they showed important structural flexibility at the carboxylate region (Supplementary Fig. 3c), suggesting that the carboxylate group is not in a catalytically competent position in the crystal structures. Moreover, the palmitate is not a substrate neither an inhibitor of the reaction catalysed by PatA (Supplementary Fig. 12). Our binary complexes correspond most likely to the hydrolysis reaction product of palmitoyl–CoA, thus one of the oxygen atoms found in the palmitate that interacts with H126 most likely come from a water molecule activated by H126. The S-C16CoA coordinates in PatA–S-C16CoA crystal structure were used to generate palmitoyl–CoA atomic coordinates by substitution of C16 atom with a carbonyl group followed by energy minimization. Docking calculations placed the Man*p* moiety attached to the 2-position of *myo*-inositol in PIM_2_ with its O6 atom favourably positioned to receive the palmitate group from palmitoyl–CoA (Fig. 8b; see Online Methods for details). We propose a model in which H126 acts as the general base to abstract a proton from the hydroxyl group at position 6 of the Man*p* ring linked to the 2-position of inositol in PIM_1_ or PIM_2_, to facilitate the nucleophilic attack on the thioester of palmitoyl–CoA (Fig. 8d). The E200 gets involved in a charge relay system with H126 and the HO6 atom from the Man*p* moiety, contributing to the appropriate structural arrangement of the imidazole ring of the histidine residue and modulating its pKa to act as a base in the first step and as an acid in the second step, providing protonic assistance to the departing CoA leaving group. It is worth noting that the H126-E200 hydrogen bond was found in a *syn* orientation relative to the carboxylate^45^. The result of the nucleophilic attack is a covalent bond between the mannose ring of PIM_1_ or PIM_2_ and palmitate. As depicted in Fig. 5b, the functional role of H126 and E200 was clearly confirmed, since their substitution by alanine completely inactivated the enzyme (Supplementary Fig. 9).

In *Cm*GPAT, the catalytic site is also located at the base of the large groove of the protein, displaying the sequence HX_4_D, a well-conserved motif among the GPAT family of acyltransferases^27^. H139 and the adjacent D144 were proposed to promote a charge-relay system to facilitate the nucleophilic attack on the thioester of the acyl–CoA^26^,^37^,^38^,^46^. In PatA, H126 occupies the same location to that observed for H139 in *Cm*GPAT. However, the aspartic acid D131 of the HX_4_D motif displays a completely different arrangement when compared with D144 (Fig. 8c). Specifically, the carboxylate group of D131 makes a strong hydrogen bond with the side chain OH of Y83, and additional electrostatic interactions with the lateral chains of W130, Y163 and R164, suggesting a structural role for this residue. In that sense, the replacement of D131 by alanine preserved 37% of the enzymatic activity for PIM_2_ (Fig. 5b; Supplementary Fig. 9). Altogether, the structural information strongly supports a common catalytic mechanism for both families of enzymes.

## Discussion

**Figure 9:**
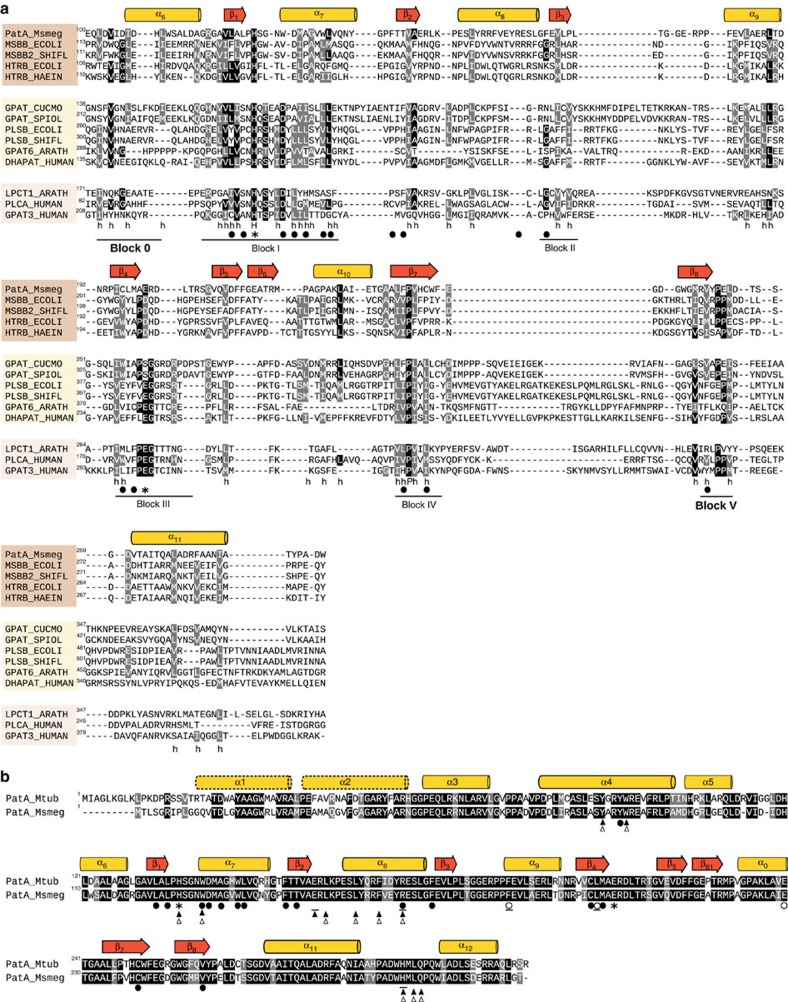
Structure weighted sequence alignment of PatA with eukaryotic/prokaryotic acyltransferases. (**a**) The protein sequences were extracted from UniProt accession numbers A0QWG5 from *M. smegmatis* (PatA_Msmeg); P24205 from *Escherichia coli* (MSBB_ECOLI); O06659 from *Shigella flexneri* (MSBB2_SHIFL); P0ACV0 from *E. coli* (HTRB_ECOLI); P45239 from *Haemophilus influenzae* (HTRB_HAEIN); P10349 from *C. moscata* (GPAT_CUCMO); Q43869 from *Spinacia oleracea* (GPAT_SPIOL); P0A7A7 PlsB from *E. coli* (PLSB_ECOLI); Q7UBC6 PlsB from *S. flexneri* (PLSB_SHIFL); O80437 from *Arabidopsis thaliana* (GPAT6_ARATH); O15228 from *Homo sapiens* (DHAPAT_HUMAN); Q8L7R3 from *Arabidopsis thaliana* (LPCT1_ARATH); Q99943 from *H. sapiens* (PLCA_HUMAN); and Q53EU6 from *H. sapiens* (GPAT3_HUMAN). Conserved positions are shown in black and grey background. Conserved hydrophobic residues are indicated with an *h*. The secondary structural elements corresponding to the 3D structure of PatA are shown above the alignment. Catalytic amino acids and those involved in palmitate binding are indicated as asterisks and black circles, respectively. (**b**) Structural similarity of *M. tuberculosis* H37Rv and *M. smegmatis* mc^2^155 PatA. Catalytic amino acids are indicated as asterisks. Amino acids involved in palmitate and pantotheinate binding are indicated as black and white circles, respectively. Residues proposed to interact with PIM_1_ and PIM_2_ based on the dockings are shown as white and black triangles, respectively. Amino acids mutated are underlined.

Amino-acid sequence alignment revealed that PatA has strong resemblance to HtrB and MsbB, two key acyltransferases involved in the biosynthesis of bacterial lipopolysaccharides (Fig. 9a)^47^,^48^. HtrB and MsbB catalyse the last steps of Kdo2-lipid A biosynthesis in Gram-negative bacteria, consecutively adding the secondary lauroyl and myristoyl residues to the distal glucosamine unit. Both enzymes prefer acyl–ACP donors but can also function with acyl–CoA substrates^7^,^8^. The fact that HtrB, MsbB and PatA preferentially use lauroyl (C12), myristoyl (C14) or palmitoyl (C16) derivatives might suggest the occurrence of a hydrocarbon ruler mechanism for acyl moieties recognition. PatA, HtrB and MsbB enzymes are distantly related to the glycerol-3-phosphate (GPAT), lysophosphatidic acid (LPAAT), dihydroacetone phosphate (DHAPAT) and 2-acylglycero-phosphatidylethanolamine (LPEAT) families of acyltransferases (Fig. 9)^5^,^27^. GPAT, LPAAT, DHAPAT, and LPEAT display highly conserved residues distributed in four regions, and named as blocks 1–4 (refs 27, 41). According to multiple sequence alignments among the HtrB, MsbB, GPAT, LPAAT, DHAPAT and LPEAT families, weighted by structural alignment of PatA and *Cm*GPAT, a common core can be defined (Fig. 9). It is worth noting that we respected the classical names of blocks 1–4 as reported in the literature, introducing two new regions as blocks 0 and 5. Critical residues and their interactions in the reaction centre are essentially preserved in all these acyltransferase families, strongly supporting a common catalytic mechanism. Interestingly, the alignment suggests that members of the GPAT, LPAAT, DHAPAT and LPEAT families display a conserved aspartate residue that participates in the charge relay system with the conserved histidine of the HX_4_D motif (block 1)^26^,^49^. In contrast, HtrB, MsbB and PatA seem to use a glutamate/aspartate residue located in block 3, suggesting a divergence among these families of acyltransferases. The structural divergence of the carboxylate group acting as a pKa modulator of the catalytic histidine residue might be due to the requirement of the acyltransferases to accommodate acceptor molecules of different nature, as observed in PatA and *Cm*GPAT. Nevertheless, the hydrophobic nature of residues involved in fatty-acid recognition is well conserved, suggesting a common binding mode.

Finally, Ac_1_PIM_2_ appears to be a metabolic end product that accumulates at high steady-state levels in the cells as well as a precursor for more polar forms of PIMs, lipomannan and LAM^10^. Interestingly, the four enzymes involved in the biosynthetis of Ac_1_PIM_2_, the phosphatidyl-*myo*-inositol synthase PgsA1 (Rv2612c in *M. tuberculosis* H37Rv), PimA (Rv2610c), PimB (Rv2188c in *Mtb* H37Rv) and PatA (Rv2611c), were found to be essential for the growth of *M. smegmatis* and/or *M. tuberculosis*^10^,^12^,^17^,^50^. Importantly, the amino-acid sequences of *M. smegmatis* and *M. tuberculosis* versions of PatA displayed 74% identity and 84% similarity (Fig. 9b). All residues that participate in the catalytic mechanism and palmitoyl binding, as well as those proposed to interact with the CoA and PIM_2_ substrates are strictly conserved between both proteins. Thus, the structural data presented here offers exciting possibilities for inhibitor design and the discovery of chemotherapeutic agents against this major human pathogen.

## Methods

### Expression and purification of PatA in *M. smegmatis*

A truncated version of PatA lacking the first 12 residues of the protein (PatA; MSMEG_2934)^28^, was purified as previously described with the following modifications. *M. smegmatis* mc^2^155 cells transformed with the corresponding plasmid pJAM2-*patA* were grown in MM63 medium (15 mM (NH4)_2_SO_4_, 10 mM KH_2_PO_4_, 18 μM FeSO_4_.7H_2_O, pH 7.0) supplemented with 1 mM MgSO_4_, 0.025% (v/v) tyloxapol and 0.2% (w/v) succinate and 20 μg ml^−1^ of kanamycin. When the culture reached OD_600_=0.6 the expression of PatA was induced by adding 0.2% acetamide. After 16 h at 37 °C, cells were collected at 4,000*g* for 10 min and resuspended in 50 mM Tris-HCl pH 7.5, 500 mM NaCl, 40 mM imidazole (solution A) containing protease inhibitors (Complete EDTA-free, Roche). The cells were resuspended in solution A (1 g of cells per 5 ml of solution A) and disrupted by sonication in 15 cycles of 60 s pulses with 90 s cooling intervals between the pulses. PatA was solubilized from the mycobacterial membrane by the addition of 2 mM CHAPS. The suspension was gently stirred during 1 h at 4 °C and centrifuged at 11,000*g* for 20 min. The supernatant was applied to a HisTrap Chelating column (1 ml, GE HealthCare) equilibrated with solution A. The column was then washed with solution A until no absorbance at 280 nm was detected. Elution was performed with a linear gradient of 40–500 mM imidazole in solution A at 1 ml min^−1^. The resulting PatA preparation displayed a single protein band when run on a 12% NuPAGE Bis–Tris precast gel stained with SimplyBlue SafeStain (Invitrogen). The purified recombinant PatA protein was stored at 4 °C and then concentrated for crystallization screening by using a Vivaspin 20 spin concentrator (Vivascience) with a 10-kDa-molecular mass cutoff.

### Site-directed mutagenesis

The PatA-H126A, PatA-D131A, PatA-E149A, PatA-R164A, PatA-E200A and PatA-H284A mutants, and double mutant PatA-F182W/L197W were synthetized by GenScript using the pJAM2-*patA* construct, and further expressed and purified to apparent homogeneity as described for the recombinant PatA enzyme.

### PatA–C16 complex crystallization and data collection

Three crystal forms were obtained, referred thereafter as PatA–C16-1, PatA–C16-2 and PatA–C16-3. The first and second crystal forms were obtained by mixing 0.25 μl of PatA at 5 mg ml^−1^ in 20 mM Tris-HCl pH 7.5 with 0.25 μl of mother liquor containing 100 mM Tris-HCl pH 7.0, 230 mM MgCl_2_ and 12–16% (w/v) PEG 8,000. Crystals grew in 7–15 days and were transferred to a cryo-protectant solution containing 25% ethylene glycol and frozen under liquid nitrogen. Complete X-ray diffraction data sets were collected at beamline I03 (Diamond Light Source, Oxfordshire, UK) and processed with XDS program^51^. The second crystal form of PatA diffracted to a maximum resolution of 2.9 Å and crystallized with four molecules in the asymmetric unit and space group *P* 2_1_ (Supplementary Table 1; PatA–C16-2). The first crystal form of PatA diffracted to a maximum resolution of 2.06 Å and crystallized with two molecules in the asymmetric unit and space group *C* 2 (Supplementary Table 1; PatA–C16-1). The third crystal form was obtained by mixing 0.25 μl of PatA at 5 mg ml^−1^ in 25 mM Tris-HCl pH 7.5, 150 mM NaCl with 0.25 μl of mother liquor containing 100 mM Tris-HCl pH 8.5 and 20% ethanol. Crystals grew in 3 days and were transferred to a cryo-protectant solution containing 25% sucrose and frozen under liquid nitrogen. A complete set of X-ray diffraction data were collected at beamline X06SA Swiss Light Source (Villigen, Switzerland) and processed with XDS program. PatA–C16-3 crystals diffracted to a maximum resolution of 2.43 Å and crystallized with one molecule in the asymmetric unit and space group *P* 4_2_ 2_1_ 2 (Supplementary Table 1).

### PatA-S-C16CoA complex crystallization and data collection

One crystal form was obtained by mixing 0.25 μl of PatA at 5.1 mg ml^−1^ in 1 mM *S*-hexadecyl Coenzyme A (S-C16CoA; stock solution at 10 mM in 20 mM Tris-HCl pH 7.5) and 20 mM Tris-HCl pH 7.5 with 0.25 μl of mother liquor containing 100 mM HEPES pH 7.5, 500 mM ammonium sulfate and 30% (v/v) 2-methyl-2,4-pentanediol. Crystals grew in 7–15 days and were directly frozen under liquid nitrogen. A complete X-ray diffraction data set was collected at beamline I03 (Diamond Light Source) and processed with XDS program^51^. PatA-S-C16CoA diffracted to a maximum resolution of 3.28 Å and crystallized with four molecules in the asymmetric unit and space group *P* 2_1_ (Supplementary Table 1).

### PatA structure determination and refinement

PatA crystals of form *C* 2 (PatA–C16-1) were soaked with 10 different platinum salts at 1 mM concentration for a time period of 130–145 min (HR2-442, Hampton Research) followed by flash freezing in liquid-N_2_. Anomalous data were collected at the theoretical L–I absorption edge of Pt (13,879.9 eV–0.8933 Å). Oscilation images were collected at I04 beamline (Diamond Light Source) with an oscillation angle of 0.2 for a total of 1,800 images using a Pilatus 6M-F pixel detector. Data were collected with an attenuated X-ray beam (5% transmission) and a 0.04-s exposure time per image. Data were integrated and scaled in XDS and experimental phases determined using the SHELXC/D/E package^52^. Data of a PatA crystal soaked with 1 mM K_2_PtCl_4_ for 130 min were used for experimental phasing with a 2.5-Å data cutoff applied, giving a mean value |Δ*F*|/*σ*(Δ*F*) in the highest resolution shell of 0.9. The substructure determination located two Pt atoms in the asymmetric unit (CC=33.64, CC(weak)=20.71 and CFOM=54.35). Experimental phases were determined and subsequently used for initial cycles of model building and density modifications by SHELXE. Buccaneer^53^ and the CCP4 suite^54^ were used for further model extention. The structure determination of PatA–C16-1, PatA–C16-2, PatA–C16-3 and PatA–S-C16CoA were carried out by molecular replacement using Phaser^55^ and the PHENIX suite^56^ and the PatA–Pt structure as model (Supplementary Table 2). Followed by cycles of manual rebuilding and refinement using Coot^57^ and phenix.refine^58^, respectively. The structures were validated by MolProbity^59^.

### Molecular docking calculations

The crystal structure of mycobacterial PatA in complex with S-C16CoA (PatA–S-C16CoA) was investigated using the structure preparation function in MOE2013.08 (ref. 60). First, S-C16CoA coordinates in PatA–S-C16CoA crystal structure (chain A) were used to generate palmitoyl–CoA atomic coordinates by substitution of C16 atom with a carbonyl group. Then the model was prepared using the Amber12EHT force field, an all-atom force field, combining two-dimensional Extended Hueckel Theory (EHT) and Amber12 force field, with Born solvation, and hydrogen atoms were added using Protonate3D function^61^,^62^. The docking site of PIM_1_ and PIM_2_ was defined by 14 residues of the PatA–S-C16CoA crystal structure. PIM_2_ structure was retrieved from the PDB (ligand code XPX) and after substitution of acyl chains with acetyl groups, the molecule was energy minimized using MOE with a 0.1 kcal mol^−1^ Å^−1^ r.m.s. gradient threshold. PIM_1_ structure was constructed by removing one mannose ring of PIM_2_. The PIM_1_ and PIM_2_ structures were submitted to conformational search using LowModeMD with default parameters in MOE2013.08 (ref. 63). The same procedure was carried out with PIM_1_, in which a mannose residue GOLD (Genetic Optimization for Ligand Docking; Cambridge Crystallographic Data Center (CCDC), version 5.2.2) was used with default genetic algorithm parameter settings^64^,^65^ for all calculations, with the exception that the search efficiency parameter was set to 200% to improve predictive accuracy by calculating the optimal number of genetic algorithm operations for the ligand due to their large flexibility. The ASP scoring function implemented in GOLD was used to rank the docked poses; this fitness function has been optimized for the prediction of ligand-binding positions^66^,^67^. PIM_1_ and PIM_2_ were docked into the PatA–palmitoyl–CoA complex. Only docking poses having a C6-hydroxyl group of the mannose closed to the thioester of palmitoyl–CoA were kept for analysis. The best solutions were assessed by their respective docking score and by visual inspection.

### PatA acyltransferase activity assay

PatA transferase activity was measured in the assay with mycobacterial membranes^28^. Briefly, *M. smegmatis* mc^2^155 cells were broken by sonication and the membrane (100,000*g* pellet) fraction was obtained by differential centrifugation. The reaction mixtures contained 250 μg of membrane proteins, 1.2 μg of purified PimA_SM_, 10 μg of purified PatA or the mutated versions, 0.1 μCi GDP-[^14^C]mannose (specific activity of 55 mCi mmol^−1^, ARC Inc.), 0.12 mM palmitoyl–CoA (Sigma-Aldrich) in DMSO with final concentration in the reaction mixture 2% (v/v), 62 μM ATP, 10 mM MgCl_2_, and 25 mM Tris-HCl pH 7.5 in the final volume of 50 μl. Reactions were incubated 100 min at 37 °C and stopped with 300 μl of CHCl_3_/CH_3_OH (2:1, by volume). The samples were left rocking 30 min at room temperature, and centrifuged at 1,500*g* for 10 min. The organic phase (bottom) was analysed by thin layer chromatography on aluminium-coated silica 60 F_254_ plates (Merck) developed in CHCl_3_/CH_3_OH/conc. NH_4_OH/H_2_O (65:25:0.5:4), and quantified by scintillation spectrometry^28^. All enzymatic activity measurements were determined in duplicates. Following the same procedure, palmitate was assayed as a possible inhibitor or substrate at different concentrations (Supplementary Fig. 12).

### PatA palmitoyl–CoA hydrolytic activity assay

The hydrolytic activity of PatA and PatA variants against palmitoyl–CoA was measured as following a methodology described for other acyltransferases^68^,^69^. A typical reaction contained 20 mM Tris-HCl pH 8.3, 0,2 mM disulfide (5,5′-dithiobis-(2-nitrobenzoic acid)) (DTNB), 0,06 mM palmitoyl–CoA and 4 μM PatA or its variants. All reactions were carried out at 37 °C in a CARY 300 Bio UV Visible/Spectrophotometer. The spectrum of the product TNB^−2^ formed after the interaction of the DTNB and the CoA liberated from the hydrolysis of the substrate palmitoyl–CoA by the enzyme, was measured continuously at 412 nm during 20 min. All enzymatic activity measurements were determined in duplicates (Fig. 5).

### Structural analysis and sequence alignment

The sequence of PatA from *M. smegmatis* (A0QWG5) was subjected to basic local alignment search tool (BLAST) and several orthologs in *Mycobacteria* sp. were found. Afterwards, they were aligned using the ClustalW server (http://www.ebi.ac.uk/Tools/msa/clustalw2/). The structure weighted sequence alignment was performed using PROMALS3D server (http://prodata.swmed.edu/promals3d/promals3d.php). For labelling the conserved and similar residues, BoxShade server was used (http://embnet.vital-it.ch/software/BOX_form.html). Structural analysis and graphics for publications were performed with PyMOL (version 0.99) and Chimera^70^.

## Additional information

**Accession codes:** Coordinates and structure factors have been deposited in the Worldwide Protein Data Bank (wwPDB) with accession codes 5F2T, 5F2Z, 5F31 and 5F34.

**How to cite this article**: Albesa-Jové, D. *et al*. Structural basis for selective recognition of acyl chains by the membrane-associated acyltransferase PatA. *Nat. Commun.* 7:10906 doi: 10.1038/ncomms10906 (2016).

## Supplementary Material

Supplementary Information Supplementary Figures 1-12 and Supplementary Tables 1-2

## Figures and Tables

**Figure 1:**
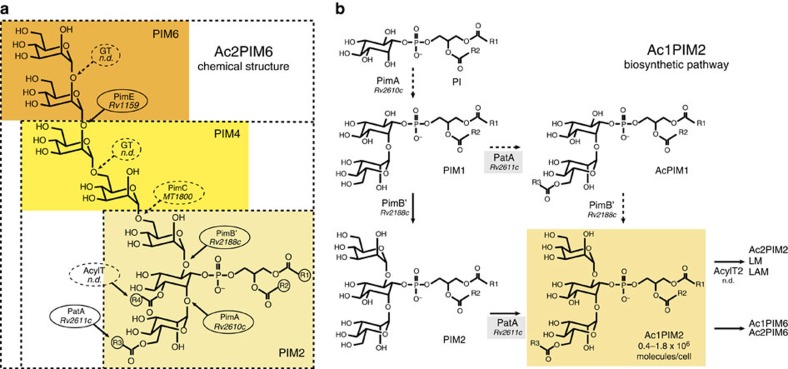
Proposed pathway for the early steps of PIM biosynthesis in mycobacteria. (**a**) Chemical structure of PIM_2/6_ and their acylated forms Ac_1/2_PIM_2_ and Ac_1/2_PIM_6_. (**b**) The two pathways originally proposed for the biosynthesis of Ac_1_PIM_2_ in mycobacteria are shown: (i) PI is mannosylated to form PIM_1_. PIM_1_ is then mannosylated to PIM_2_, which is acylated to form Ac_1_PIM_2_; (ii) PIM_1_ is first acylated to Ac_1_PIM_1_ and then mannosylated to Ac_1_PIM_2_. Our experimental evidence indicates that although both pathways might co-exist in mycobacteria, the sequence of events PI→PIM_1_→PIM_2_→Ac_1_PIM_2_ is favoured. As an important part of the literature concerning PIMs studies refers to the nomenclature based on the *Mtb* H37Rv sequences, we also include the Rv numbers to identify the proteins.
