# Supplementary material for: Structural basis for selective recognition of acyl chains by the membrane-associated acyltransferase PatA
Source: Nat Commun. 2016 Mar 11;7:10906. doi: 10.1038/ncomms10906 (PMC4792965; doi:10.1038/ncomms10906)
Supplement: Supplementary Information — Supplementary Figures 1-12 and Supplementary Tables 1-2 [file ncomms10906-s1.pdf]

a

```
MPEVVFGSVTDLGYAAGWRLVRAMPEAMAQGVFGAGARYAARNGGPEQLRRNLARVVGKPPADV
PDDLIRASLASYARYWREAFRLPAMDHGRLGEQLDVIDIDHLWSALDAGRGAVLALPHSGNWDM
AGVWLVQNYGPFTTVAERLKPESLYRRFVEYRESLGFEVLPLTGGERPPFEVLAERLTDNRPIC
LMAERDLTRSGVQVDFFGEATRMPAGPAKLAIETGAALFPVHCWFEGDGWGMRVYPELDTSSGD
VTAITQALADRFAANIATYPADWHMLQPQWIADLSDERRARLGTSRHHHHHH
```

b

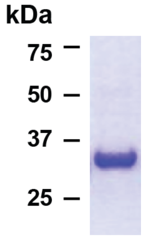

Supplementary Figure 1. Recombinant production of PatA. Amino acid sequence of PatA construct used for production in *M. smegmatis*. **(a)** The recombinant PatA construct lacks the first 12 residues of the protein. **(b)** SDS-PAGE showing purified PatA.

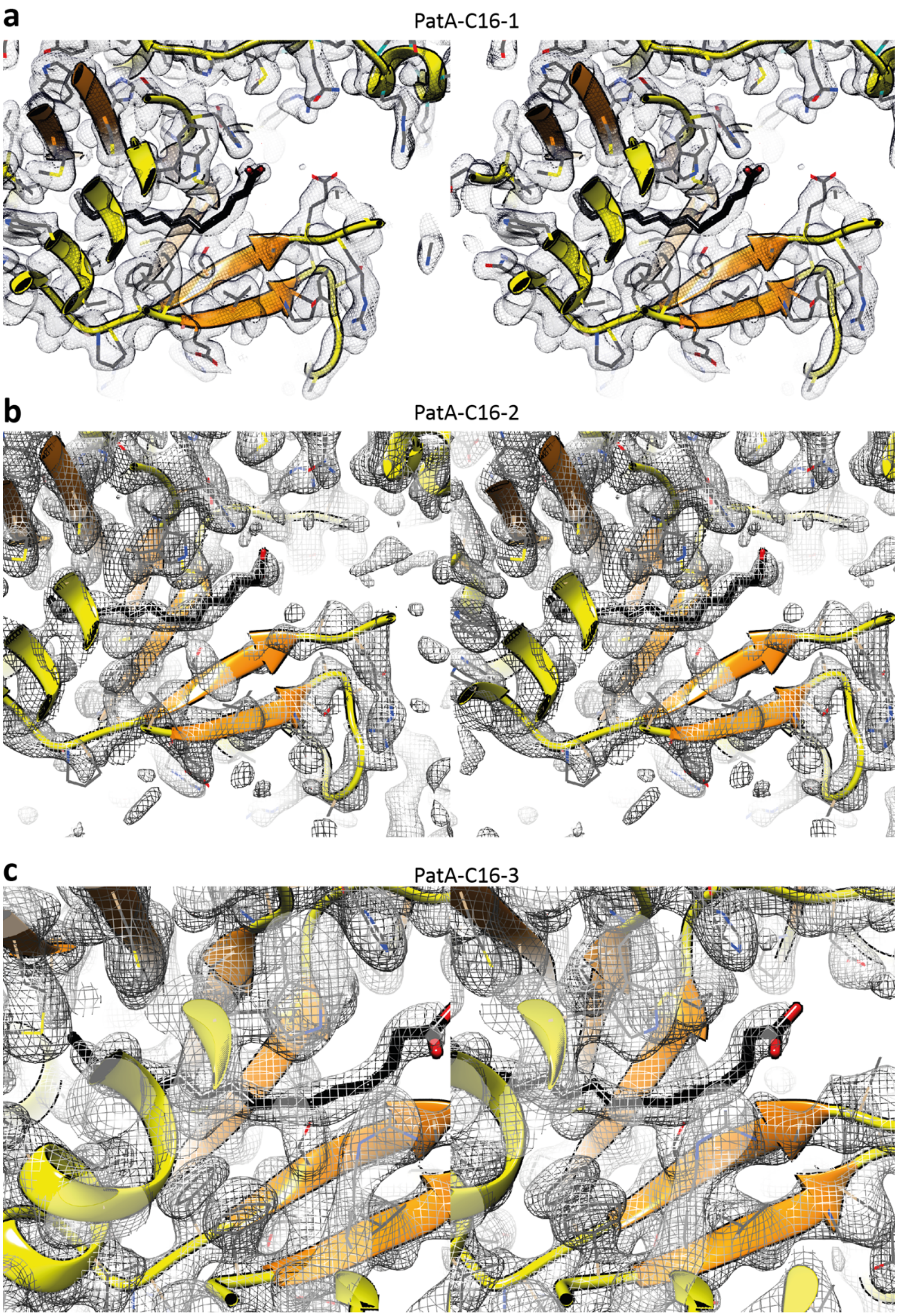

Supplementary Figure 2. Electron density maps of the refined PatA complexes in the presence of palmitate. Stereo views of the final electron density maps (2mFo-DFc contoured at 1σ) corresponding to the PatA-C16-1 (a,b), PatA-C16-2 (c,d) and PatA-C16-3 (e,f) structures.

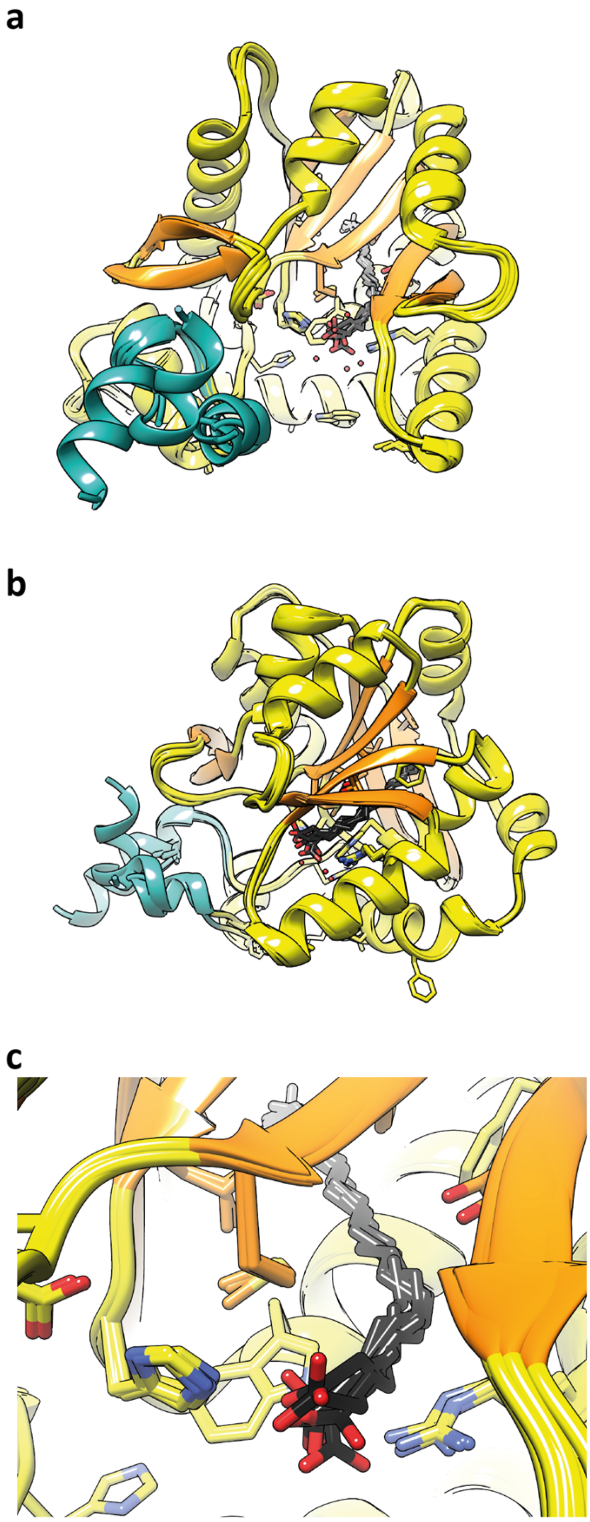

Supplementary Figure 3. Structural flexibility at the N- and C-terminus of PatA-C16-1, -2 and -3 complexes. (a,b) Cartoon representation showing the superimposed monomers obtained from crystal structures of PatA-C16-1, PatA-C16-2, and PatA-C16-3. The N- and C-terminus are colored blue. **(c)** Structural superposition of the palmitate molecules as visualized in the PatA-C16-1, PatA-C16-2, and PatA-C16-3 structures.

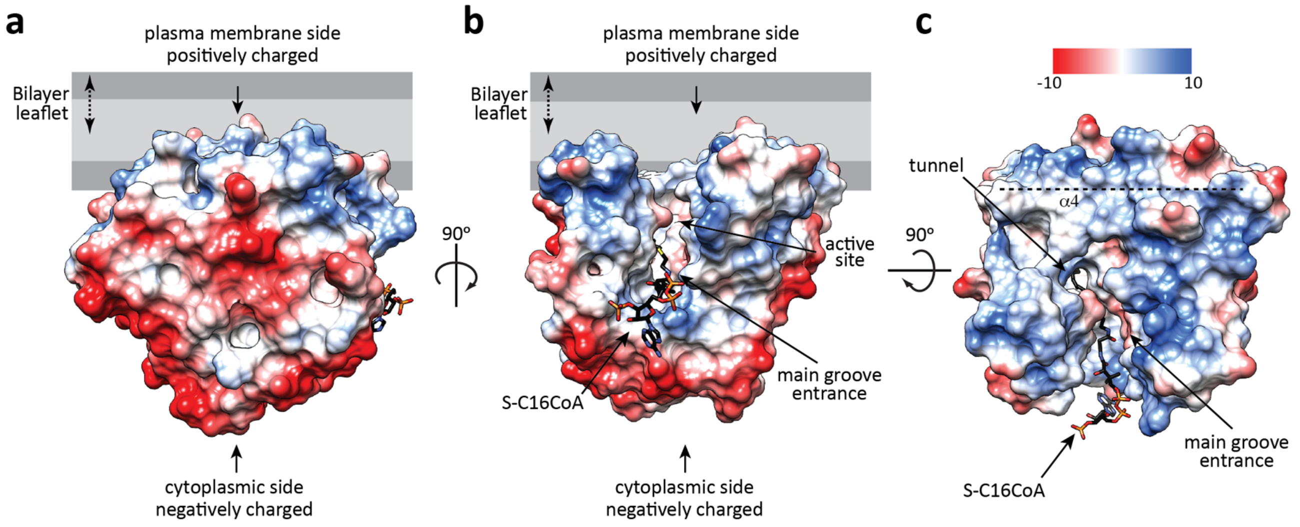

Supplementary Figure 4. Membrane association of PatA. Electrostatic surface representation of PatA. **(a)** PatA is a polar protein, with hydrophobic and positively charged residues oriented towards the plasma membrane. Negatively charged residues are facing the cytosol. **(b,c)** The active site of the protein is located into the main groove, in close proximity to hydrophobic and positively charged residues at the membrane interface. The electrostatic potential surface has been computed by solving the Poisson-Boltzmann equation, as implemented in APBS[39], for the crystal structure of PatA in complex with S-C16CoA. The Poisson-Boltzmann electrostatic color map spans from red to blue, ranging from -10 to +10 kcal/mol.

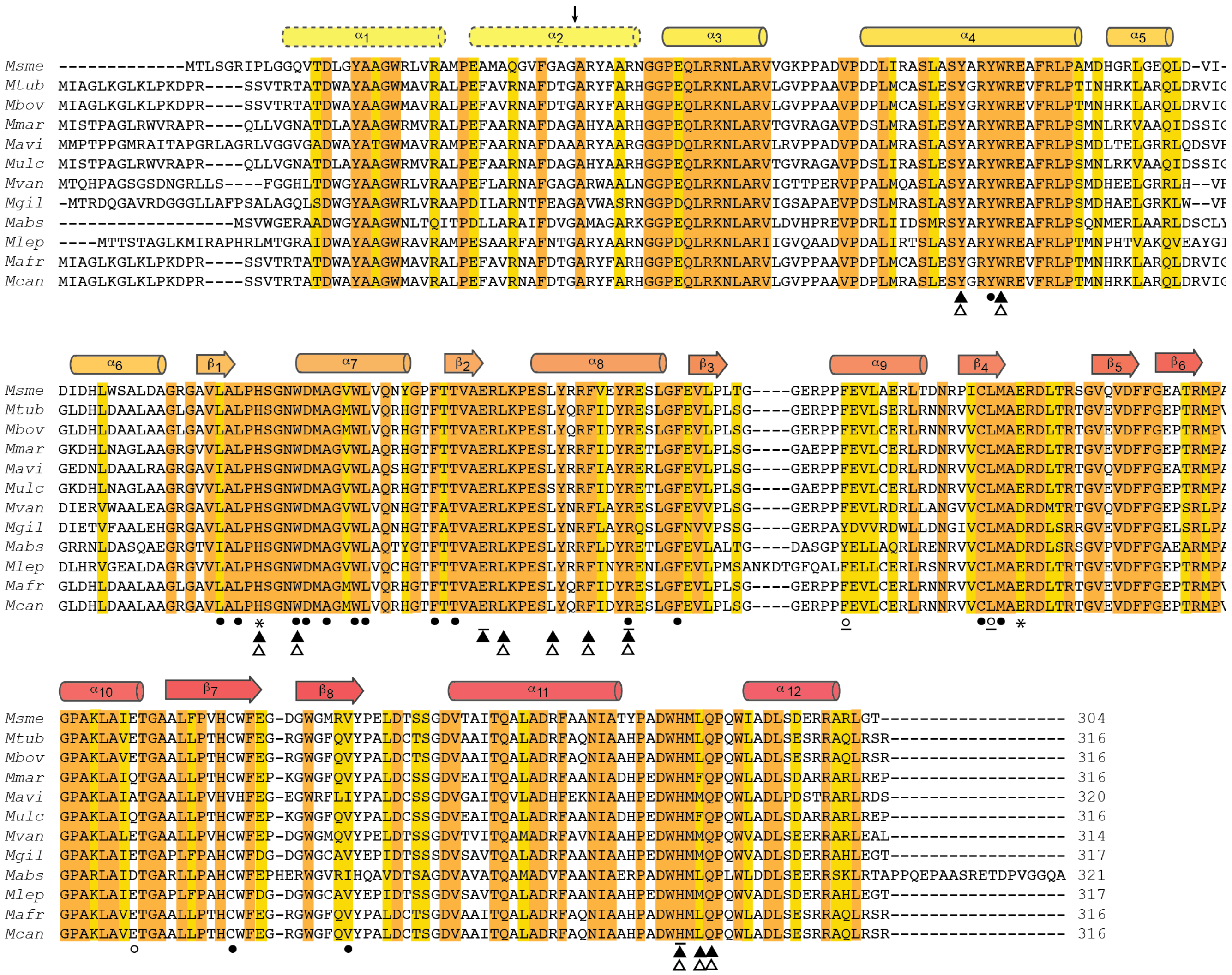

Supplementary Figure 5. Multiple sequence alignment of PatA with mycobacterial orthologs. Orthologs shown are from *Mycobacterium smegmatis* (*Msme*, A0QWG5), *M. tuberculosis* (*Mtub*, O06203), *M. bovis* (*Mbov*, Q7TY87), *M. marinum* (*Mmar*, B2HN43), *M. avium* (*Mavi*, AIV25898.1), *M. ulcerans* (*Mulc*, ABL05460.1), *M. vanbaalenii* (*Mvan*, ABM13371.1), *M. gilvum* (*Mgil*, ADT99794.1), *M. abscessus* (*Mabs*, AIV11722.1), *M. leprae* (*Mlep*, Q7AQJ0), *M. africanum* (*Mafr*, F8M2W4), and *M. canettii* (*Mcan*, L0PY90). Strictly conserved positions are shown in orange background. The secondary structural elements corresponding to the three distinct 3D structures of PatA are shown below the alignment. Catalytic amino acids are indicates as asterisks. Amino acids involved in palmitate and pantotheinate binding are indicated as black and white circles, respectively. Residues proposed to interact with PIM_1 and PIM_2 based on the dockings are shown as white and black triangles, respectively. Amino acids mutated are underlined.

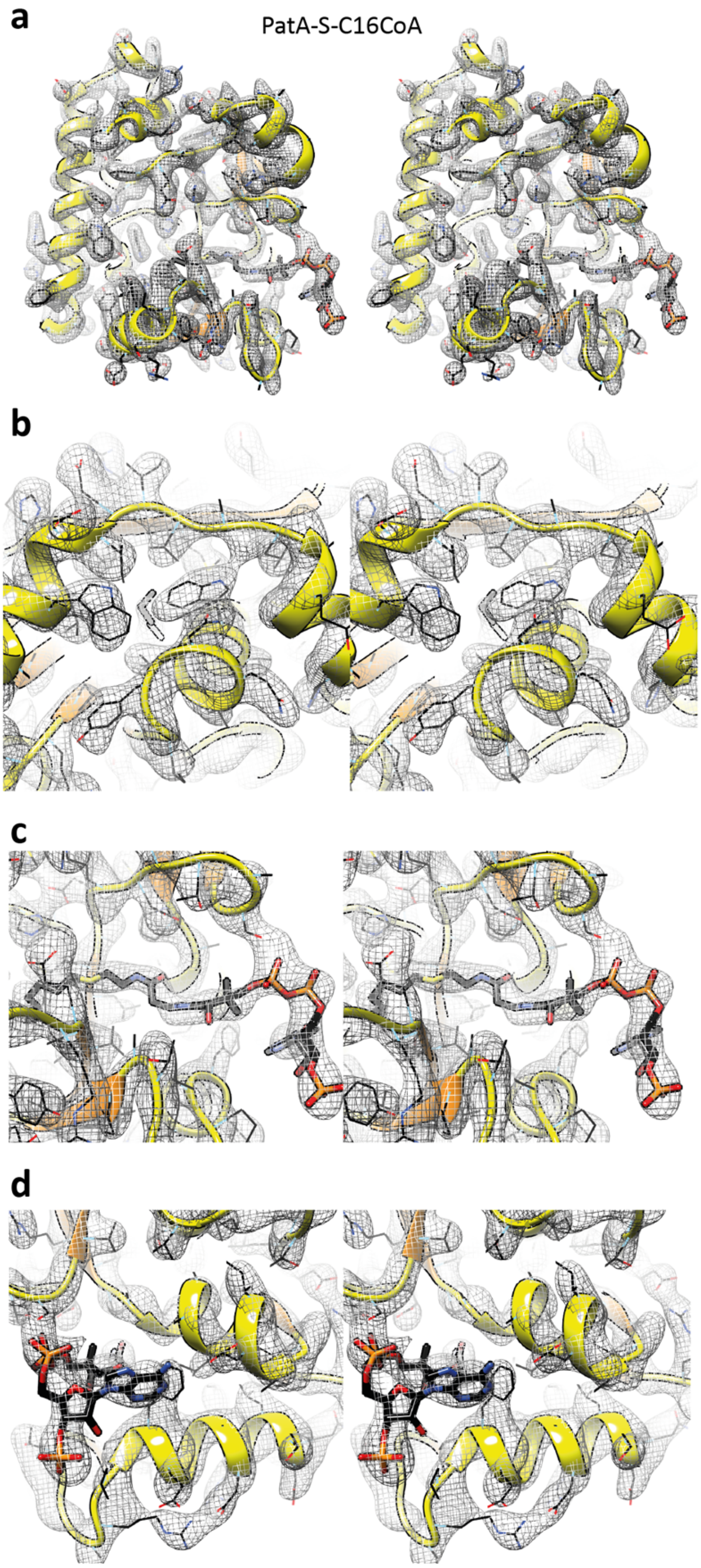

Supplementary Figure 6. Electron density maps of the refined PatA complex in the presence of S-C16CoA. **(a,b)** Stereo views of the final electron density maps (2mFo-DFc contoured at 1σ) corresponding to a selected region of the PatA-S-C16CoA structure (reference chain A). **(c,d)** Stereo views of the final electron density maps (2mFo-DFc contoured at 1σ) corresponding to a selected region of the PatA-S-C16CoA structure, in which the S-C16CoA molecule is highlighted (reference chain A).

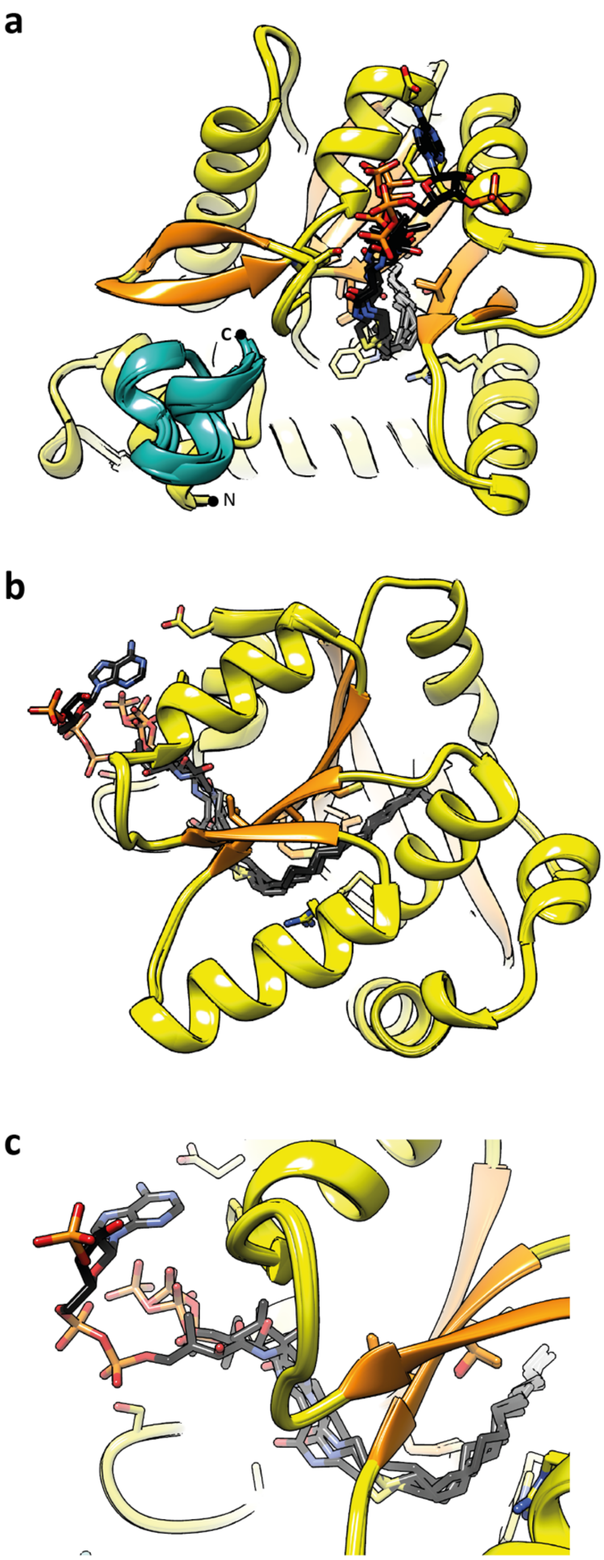

Supplementary Figure 7. Structural flexibility of S-C16CoA as visualized in the PatA-S-C16CoA complex. **(a,b)** Cartoon representation showing the four superimposed monomers obtained from the crystal structure PatA-S-C16CoA. N- and C-terminus are indicated. **(c)** Close view of the S-C16CoA binding site in PatA. Both palmitoyl and pantetheine moieties binding to PatA are conserved in all four monomers in the asymmetric unit. The phosphorylated ADP of S-C16CoA was visible in one molecule of the asymmetric unit, exposed to the bulk solvent.

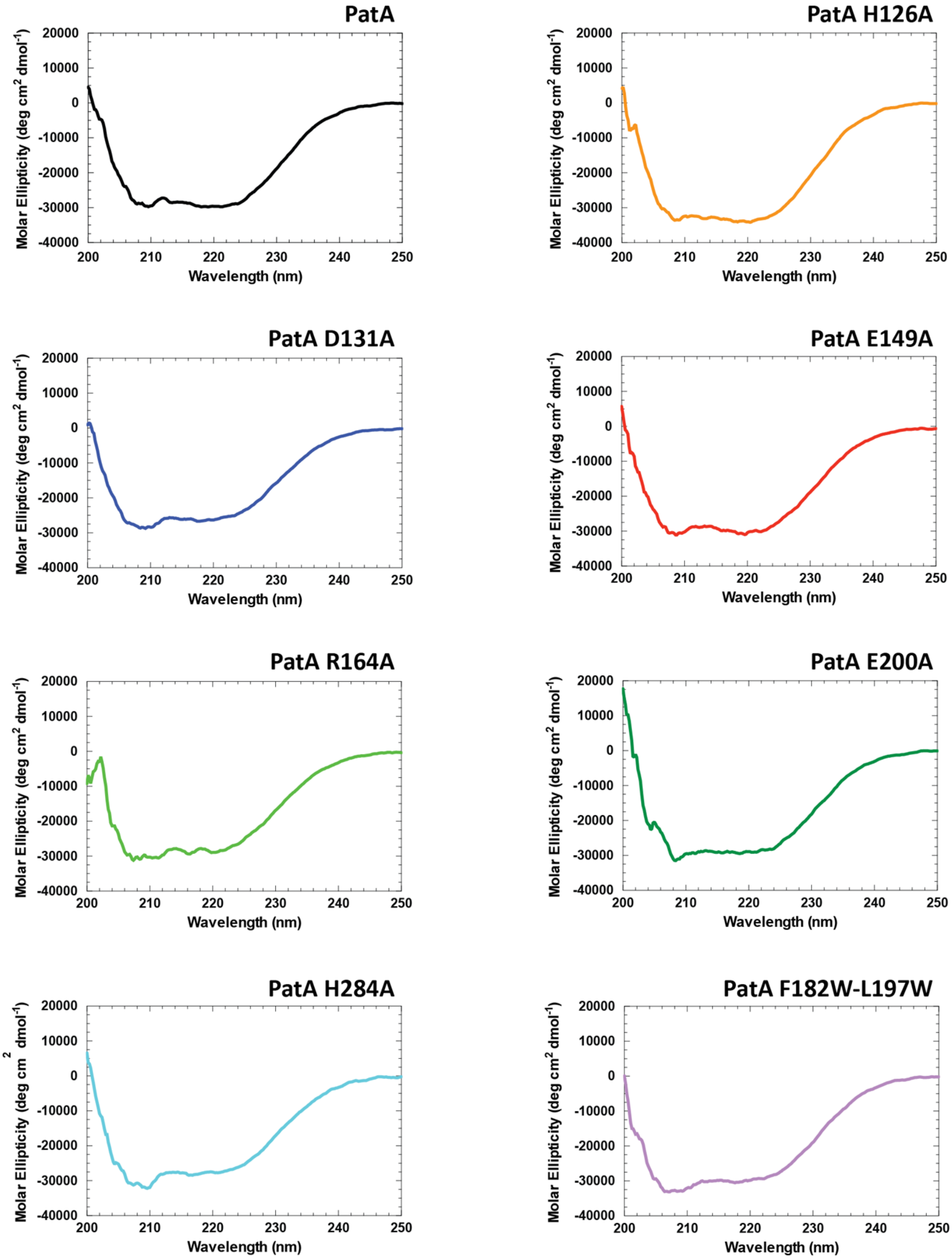

Supplementary Figure 8. Far-UV CD profile of PatA and PatA variants. The far-UV CD of PatA and PatA variants were recorded at 20 °C (see ‘Online Methods’ for details).

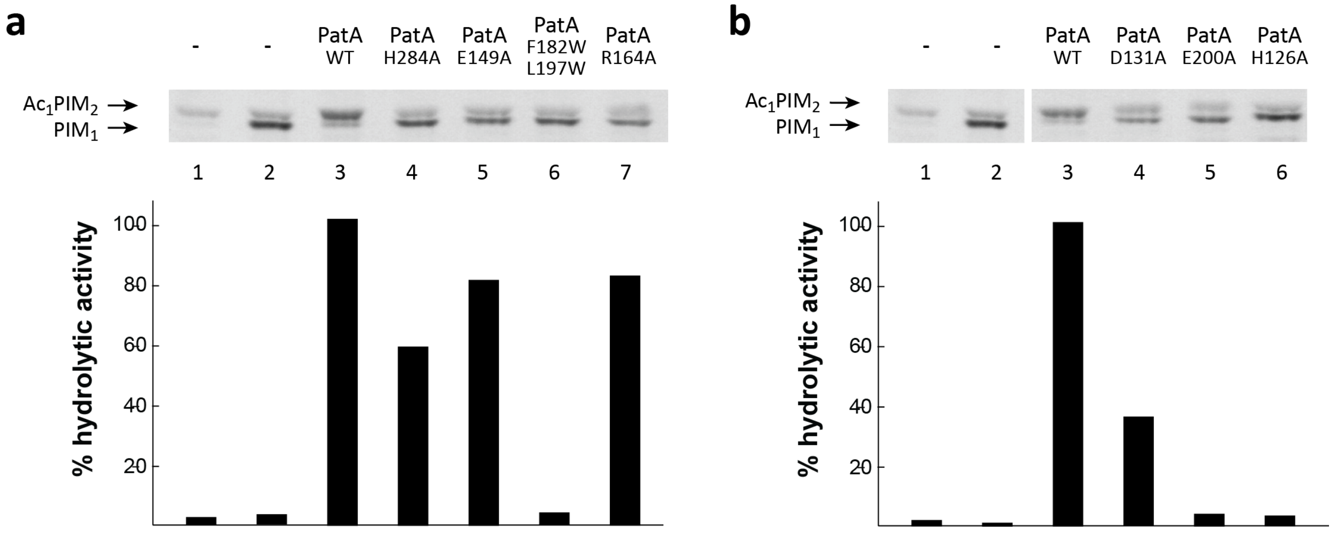

Supplementary Fig. 9. Duplicate enzymatic activity measurements of selected PatA variants. (a) In the upper panel, the acyltransferase activity of purified PatA and variants involved in substrate binding are shown. Reaction mixtures contained crude membranes from *M. smegmatis* mc^{2}155 and GDP-[14C]-mannose as a tracer (lane 1), supplemented with PimA (lanes 2 to 7) and palmitoyl-CoA (lanes 3 to 7) and purified PatA (lane 3), PatA-H284A (lane 4), PatA-E149A (lane 5), double mutant PatA-F182W/L197W (lane 6) and PatA-R164A (lane 7). The lipids were extracted from reaction mixtures and analysed by TLC and autoradiography as described in Methods section. In the lower panel, the hydrolytic activity against palmitoyl-CoA is shown. (b) In the upper panel, the acyltransferase activity of purified PatA and variants involved in catalysis are shown (see Supplementary Fig. 9 for duplicates). Reaction mixtures contained crude membranes from *M. smegmatis* mc^{2}155 and GDP-[14C]-mannose as a tracer (lane 1), supplemented with PimA (lane 2 to 6) and palmitoyl-CoA (lanes 3 to 6) and purified PatA (lane 3), PatA-D131A (lane 4), PatA-E200A (lane 5) and PatA-H126A (lane 6). In the lower panel, the hydrolytic activity against palmitoyl-CoA is shown.

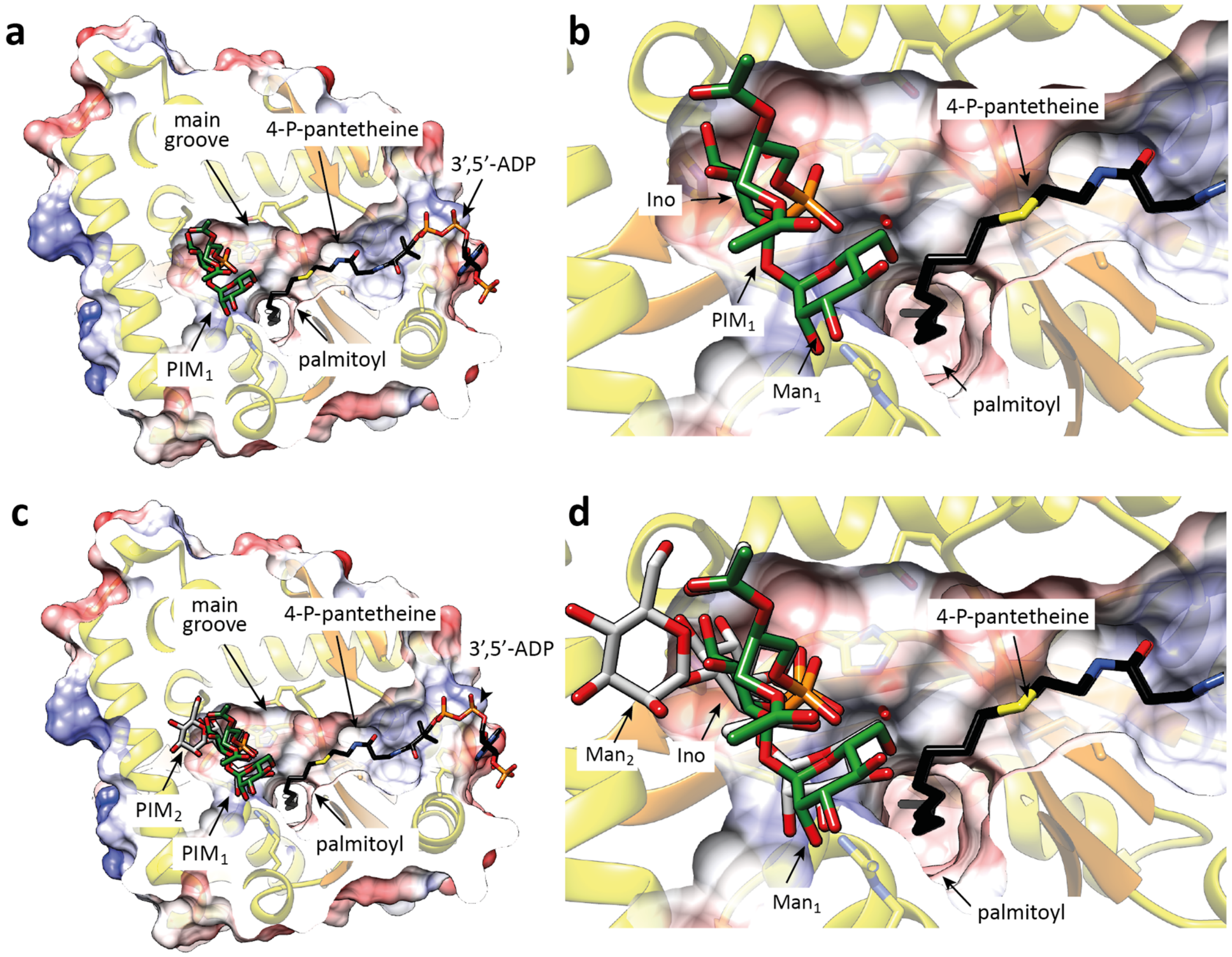

Supplementary Figure 10. Structural comparison of the PIM$_1$ and PIM$_2$ binding sites. (a,b) Two views showing the docking calculations in which PIM$_1$ (green) attaches to the end side of the main groove, and close to the hydrophobic tunnel. **(c,d)** Two views showing the comparison of the docking calculations for PIM$_1$ and PIM$_2$ (grey).

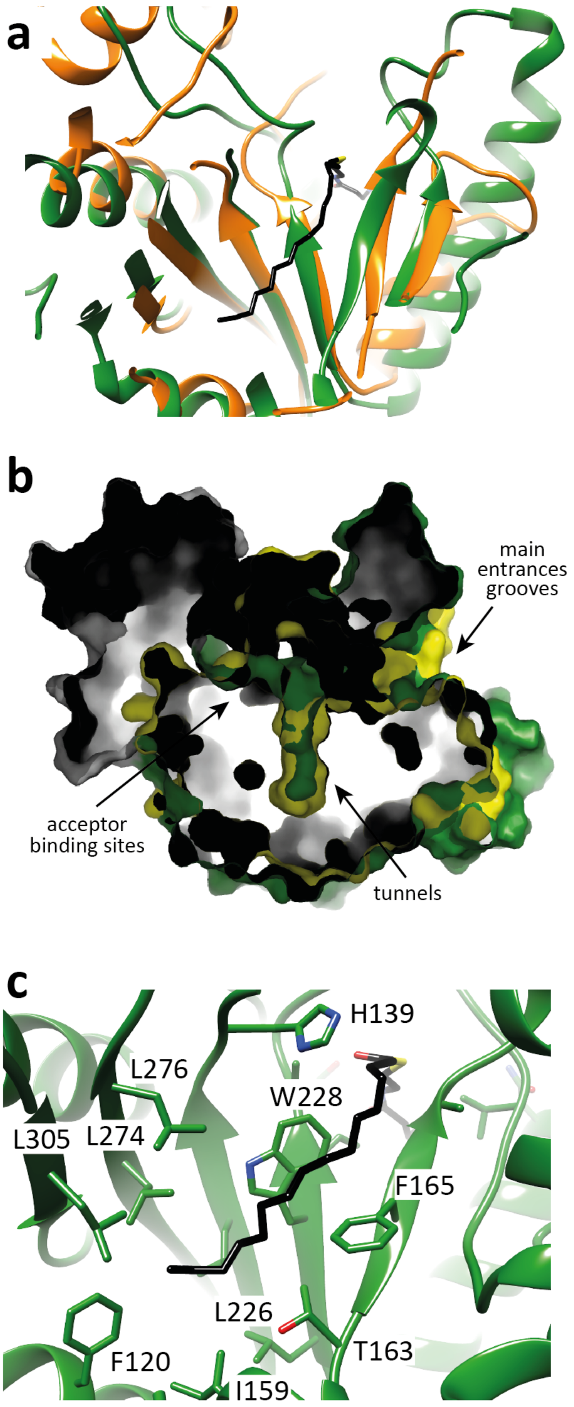

Supplementary Figure 11. Structural similarity between PatA and *Cm*GPAT. (a) Structural superposition of the β-sheet core of PatA (orange) and that of the *Cm*GPAT (green). **(b)** Structural superposition of the acyl binding tunnels in PatA (yellow) and *Cm*GPAT (green). **(c)** Localization of the fatty acid binding site in *Cm*GPAT as revealed by docking calculations.

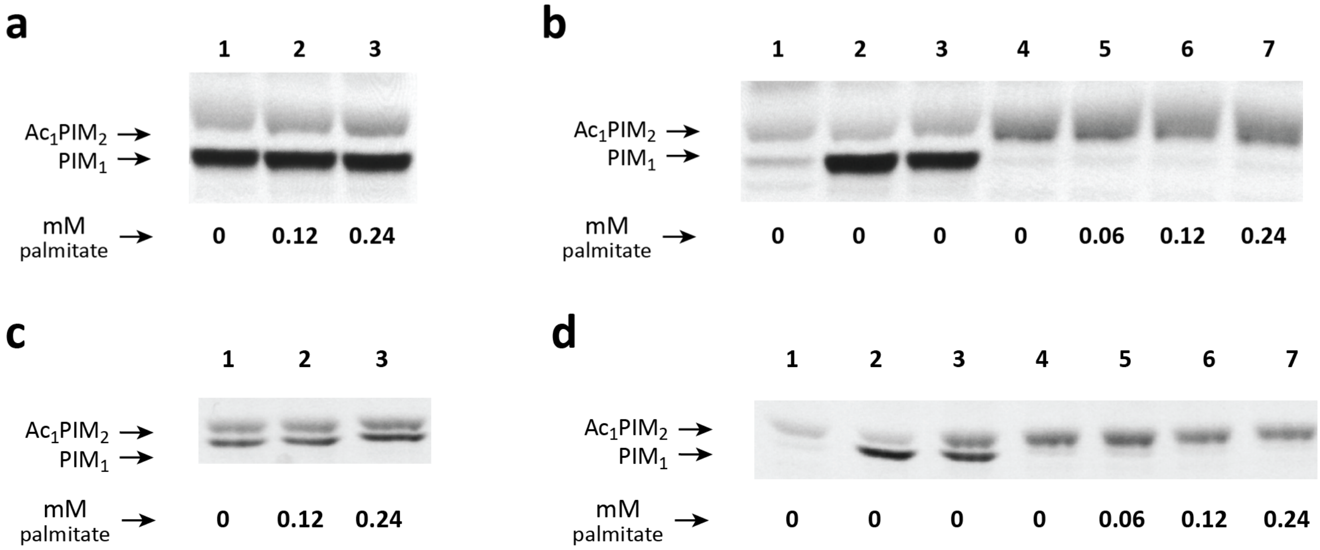

Supplementary Figure 12. Palmitate is neither a substrate or inhibitor of PatA. (a) Reaction mixtures contained 250 μg of crude membranes from *M. smegmatis* mc^2155 and 0.1 μCi GDP-[^{14}C]-mannose as a tracer supplemented with purified PimA and PatA (lane 1 to 3), and 120 μM (lane 2) and 240 μM (lane 3) palmitate. The lipids were extracted from reaction mixtures and analyzed by TLC and autoradiography as described in Methods section. **(b)** Reaction mixtures contained 250 μg crude membranes from *M. smegmatis* mc^2155 and 0.1 μCi GDP-[^{14}C]-mannose as a tracer (lane 1), supplemented with purified PimA (lanes 2 to 7), purified PatA (lanes 3 to 7), 120 μM palmitoyl-CoA (lanes 4 to 7), and palmitate at 60 μM, 120 μM and 240 μM, respectively. **(c,d)** Duplicates of enzymatic activity measurements displayed in (a) and (b) respectively.

Supplementary Table 1: Data collection and refinement statistics.

	PatA-C16-1	PatA-C16-2	PatA-C16-3	PatA-S-C16CoA
**Beamline**	I03 (DLS)	I03 (DLS)	PXI-X06SA (SLS)	I03 (DLS)
**Wavelength (Å)**	0.97625	0.97625	0.99996	0.978000
**Resolution range (Å)**	56.60 – 2.06	92.37 – 2.90	40.19 – 2.43	47.12 - 3.28
**Space group**	*C* 2	$P\,2_1$	$P\,4_2\,2_1\,2$	$P\,2_1$
**Unit cell**	93.60 71.09 76.2<br>90 91.95 90	70.83 84.48 98.80<br>90 110.78 90	80.30 80.30 113.77<br>90 90 90	81.08 80.27 97.38<br>90 110.92 90
**Total reflections**	96616 (4273)	73942 (5870)	153438 (11538)	60657 (5816)
**Unique reflections**	30533 (1973)	23420 (1759)	14652 (1057)	17931 (1708)
**Multiplicity**	3.2 (2.2)	3.2 (3.3)	10.5 (10.9)	3.4 (3.4)
**Completeness (%)**	98.5 (86.7)	96.2 (99.2)	100.0 (100.0)	99.13 (94.36)
**Mean I/sigma(I)**	10.6 (2.0)	6.6 (1.7)	4.6 (1.1)	11.18 (1.47)
**Wilson B-factor**	21.81	54.57	41.10	103.69
**R-sym**	0.068 (0.410)	0.115 (0.508)	0.122 (0.669)	0.091 (0.898)
**R-factor**	0.1714 (0.2609)	0.2389 (0.3409)	0.2236 (0.2854)	0.2640 (0.4051)
**R-free**	0.2320 (0.3323)	0.2737 (0.4251)	0.2793 (0.3599)	0.3060 (0.4495)
**Number of non-H atoms**	4441	7720	2098	7597
**Macromolecules**	4055	7548	1989	7400
**Ligands**	37	72	22	174
**Water**	349	100	87	23
**Protein residues**	509	1003	256	1011
**RMS(bonds)**	0.004	0.005	0.003	0.004
**RMS(angles)**	0.90	0.99	0.76	1.08
**Ramach. favored (%)**	99	99	97	99
**Ramach. outliers (%)**	0	0	0.39	0
**Clashscore**	1.49	2.89	1.01	2.46
**Average B-factor**	20.10	36.60	53.70	111.90
**Macromolecules**	19.40	36.90	53.90	111.30
**Ligands**	26.00	23.30	55.90	139.40
**Solvent**	26.40	20.60	46.60	86.30

Supplementary Table 2: Data collection for experimental phasing

	**PatA•Pt**
**Beamline**	I04 (DLS)
**Wavelength (Å)**	0.8933
**Photon energy (keV)**	13.8799
**Exposure (s)**	0.040
**Transmission (%)**	5.01
**Beamsize (μm)**	90 x 45
**Total images**	1800
**Ω Osc (°)**	0.20
**Resolution range (Å)**	78.09 - 1.88
**SigAno[#] > 0.88 (Å)**	78.09 – 2.33
**Space group**	*C* 2
**Unit cell**	95.99 71.04 78.200<br>90 93.01 90
**Total reflections**	254696 (8905)
**Unique reflections**	41213 (2343)
**Multiplicity**	6.2 (3.8)
**Completeness (%)**	96.4 (74.6)
**Mean I/sigma(I)**	15.0 (2.1)
**Wilson B-factor**	20.44
**R-sym**	0.076 (0.462)

[#] Mean anomalous difference in units of its estimated standard deviation (|F(+)-F(-)|/Sigma).
